# Neutrophil infiltration associated genes on the prognosis and tumor immune microenvironment of lung adenocarcinoma

**DOI:** 10.3389/fimmu.2023.1304529

**Published:** 2023-12-22

**Authors:** Renwang Liu, Guangsheng Zhu, Yonglin Sun, Mingbiao Li, Zixuan Hu, Peijun Cao, Xuanguang Li, Zuoqing Song, Jun Chen

**Affiliations:** ^1^ Department of Lung Cancer Surgery, Tianjin Medical University General Hospital, Tianjin, China; ^2^ Tianjin Key Laboratory of Lung Cancer Metastasis and Tumour Microenvironment, Lung Cancer Institute, Tianjin Medical University General Hospital, Tianjin, China; ^3^ Gynecology and Obstetrics Department, Tianjin Third Central Hospital, Tianjin, China

**Keywords:** neutrophil infiltration, tumor associated neutrophil, tumor immune microenvironment, LUAD, bioinformatics analysis, survival analysis

## Abstract

The neutrophils exhibit both anti-tumor and pro-tumor effects in cancers. The correlation between neutrophils and tumor development in lung adenocarcinoma (LUAD) is still uncertain, possibly due to a lack of specific neutrophil infiltration evaluation methods. In this study, we identified 30 hub genes that were significantly associated with neutrophil infiltration in LUAD through data mining, survival analysis, and multiple tumor-infiltrating immune cells (TICs) analysis, including TIMER, CIBERSORT, QUANTISEQ, XCELL, and MCPCOUNTER. Consensus clustering analysis showed that these 30 hub genes were correlated with clinical features in LUAD. We further developed a neutrophil scoring system based on these hub genes. The neutrophil score was significantly correlated with prognosis and tumor immune microenvironment (TIME) in LUAD. It was also positively associated with PD-L1 expression and negatively associated with tumor mutational burden (TMB). When combined with the neutrophil score, the predictive capacity of PD-L1 and TMB for prognosis was significantly improved. Thus, the 30 hub genes might play an essential role in the interaction of neutrophils and LUAD, and the neutrophil scoring system might effectually assess the infiltration of neutrophils. Furthermore, we verified the expression of these 30 genes in the LUAD tumor tissues collected from our department. We further found that overexpressed TNFAIP6 and TLR6 and downregulated P2RY13, SCARF1, DPEP2, PRAM1, CYP27A1, CFP, GPX3, and NCF1 in LUAD tissue might be potentially associated with neutrophils pro-tumor effects. The following *in vitro* experiments demonstrated that TNFAIP6 and TLR6 were significantly overexpressed, and P2RY13 and CYP27A1 were significantly downregulated in LUAD cell lines, compared to BEAS-2B cells. Knocking down TNFAIP6 in A549 and PC9 resulted in the upregulation of FAS, CCL3, and ICAM-1, and the downregulation of CCL2, CXCR4, and VEGF-A in neutrophils when co-culturing with the conditioned medium (CM) from LUAD cells. Knocking down TNFAIP6 in LUAD also led to an elevated early apoptosis rate of neutrophils. Therefore, overexpressed TNFAIP6 in LUAD cancer cells might lead to neutrophils “N2” polarization, which exhibited pro-tumor effects. Further research based on the genes identified in this pilot study might shed light on neutrophils’ effects on LUAD in the future.

## 1 Introduction

Neutrophils are humans’ most abundant innate immune cells, accounting for 50-70% of all leukocytes (1, 2). It mainly participates in host defense through phagocytosis, degranulation and release of proteases, secretion of various chemokines and cytokines, and forming neutrophil extracellular traps (NETs) via NETosis to resist the invasion and reproduction of pathogenic bacteria (3, 4). The neutrophils can also be recruited and infiltrated into tumor microenvironments as tumor-associated neutrophils (TANs) (5, 6).

TANs have emerged as significant prognostic biomarkers in various cancers, such as bronchioloalveolar and renal carcinoma (7–9). It plays an essential role in tumor development and presents significant heterogeneity. On the one hand, TANs may promote tumor occurrence by generating reactive oxygen species (ROS) (10), releasing neutrophil elastase (NE) to accelerate tumor growth (11), secreting matrix metalloproteinase-9 (MMP-9) to induce angiogenesis (12), and forming NETs to facilitate tumor metastasis (13). On the other hand, TANs also exhibit anti-tumor properties, including direct killing of nascent tumor cells (14), releasing Arg1 to stimulate TRAIL expression and induce tumor cell apoptosis (15), and recruiting and activating T cells for tumor cell eradication (16, 17).

In non-small cell lung cancer (NSCLC), the abundance of neutrophils holds prognostic significance. Patients with higher neutrophil-to-lymphocyte ratios (NLR) exhibited reduced progression-free survival (PFS) and overall survival (OS) (18, 19). Early-stage NSCLC patients with heightened CD66b-positive neutrophil infiltration faced an increased likelihood of postoperative recurrence (20). However, in different subtypes of NSCLC, TANs demonstrated distinct roles (21). For instance, *Mehrdad Rakaee* et al. found that the proportion of CD66b-positive TANs presented opposing prognostic impacts between squamous cell carcinoma (SCC) and adenocarcinoma (ADC) (22). *Xinyan Liu* et al. also found that the TANs infiltration did not correlate with prognosis in lung adenocarcinoma (LUAD) (23).

These unclear effects of TANs in LUAD may be due to a lack of specific methods for assessing TANs. The underlying mechanisms of TANs’ effect on LUADs also remain unknown. Thus, in this study, we identified 30 hub genes closely associated with neutrophil infiltration in LUADs using bioinformatics approaches. Then, by using these hub genes, we constructed a specific LUADs’ TANs infiltration scoring system and analyzed its correlation with prognosis and tumor immune microenvironment. This pilot study might provide valuable insights into exploring TANs effects on LUADs.

## 2 Materials and methods

### 2.1 Data acquisition and differentially expressed genes analysis

All LUAD patients’ data, including gene expression and clinical pathological features, were downloaded from The Cancer Genome Atlas (TCGA) database. The neutrophils-specific expressed genes were downloaded from The Human Protein Atlas (THPA) database (https://www.proteinatlas.org/). Gene microarray data and clinical information of 181 tumor samples in external validation were obtained from the Gene Expression Omnibus (GEO) dataset (https://www.ncbi.nlm.nih.gov/geo/query/acc.cgi?acc=GSE50081) (24). The log2(x+0.001) transforming was performed in each expression value. R software (version 3.6.4) was used to analyze differential expression and clinical characteristics.

### 2.2 Tumor-infiltrating immune cells analysis

Five independent TICs analysis methods, including TIMER (25), MCPCOUNTER (26), XCELL (27), CIBERSORT (28), and QUANTISEQ (29), were performed to assess neutrophil infiltration. The tumor-associated immune comprehensive score was assessed via ImmunoPhenoScore (IPS) in R package IOBR (version 0.99.9) (30).

### 2.3 Neutrophil scoring construction and clustering analysis

The principal component analysis (PCA) algorithm was used for establishing neutrophil scoring according to the selected 30 hub genes. The formula was: Neutrophil_score=∑PC1_i_+PC2_i_. The consensus clustering analysis was performed via the ConsensusClusterPlus package in R software.

### 2.4 Survival analysis

The bioinformatics survival analysis was performed as previously described (31). Briefly, the CoxPH in R software was used for univariate Cox regression analysis to screen genes and to establish the Cox proportional hazards regression model. MaxStat in R was used to calculate the best cut-off value and survfit in R to analyze the differences in OS and PFS between each group.

### 2.5 Mutations and tumor mutational burden

All the level 4 Simple Nucleotide Variation datasets in TCGA were downloaded from GDC (https://portal.gdc.cancer.gov/) (32) and processed by MuTect2. The alterations were analyzed in both high and low neutrophil score groups. The TMB was calculated by the tmb function from the R package maftools (version 2.8.05).

### 2.6 Tissue specimens and qPCR

Ten fresh lung adenocarcinoma specimens with paired adjacent normal tissue samples were collected from Tianjin Medical University General Hospital between May 2023 and June 2023. The basic information of these ten patients was listed in **Supplementary Table 1**. The expression of the 30 hub genes in both cancer and normal tissue was detected by qPCR. The procedure of qPCR was described previously (33). Briefly, TRIzol Reagent (Invitrogen, USA) was used for total RNA extraction. Power SYBR Green PCR Master Mix (Applied Biosystems, USA) was used for the reaction after reverse transitions. The primer sequences in this study were listed in **Supplementary Table 2**.

### 2.7 Cell culture and transfection

The cell culture and transfections were performed as described previously (31). All the cell lines were purchased from the American Tissue Culture Collection (ATCC). The cells were maintained in RPMI 1640 medium (Gibco, USA). The si-TNFAIP6 (SIGS0003862-1), si-TLR6 (SIGS0000949-1), and si NCs (siN0000001-1-5) were purchased from RiboBio (Guangzhou, China). The si-RNA or si-NC was transfected into cells by Lipofectamine 2000 (Invitrogen, United States) under the manufacturer’s instructions.

### 2.8 Immunohistochemistry staining

IHC staining was performed as described previously (34). Briefly, the tissue slices underwent deparaffinization, followed by antigen retrieval in 5 mM Tris-HCl for 10 mins using microwave pretreatment. The 3% H2O2 was used to quench endogenous peroxidase activity, and the non-specific binding sites were blocked by serum. After incubated with anti-TNFAIP6 primary antibody (1:200, Proteintech, China) at 4°C overnight, the slides were washed and followed by horseradish peroxidase (HRP) labeled secondary antibody incubation for 30 mins at room temperature. Then, the slides were stained with diaminobenzidine and counterstained with hematoxylin. All stained slides were scanned by the Pannoramic MIDI (3DHISTECH, Hungary) and visualized in CaseViewer2.4 software (3DHISTECH, Hungary). The mages were scored automatically by Aipathwell software (Servicebio, Wuhan, China).

### 2.9 Neutrophils isolation

The peripheral blood was collected from healthy volunteers in EDTA-coated tubes. The isolation of neutrophils was using Polymorphprep (Axis-Shield, UK) under the manufacturer’s instructions. Fast Giemsa Stain Kit (Yeasen, China) was used to determine the purity of the isolated neutrophils. The neutrophils were maintained in RPMI 1640.

### 2.10 Neutrophil polarization detection

The cancer cells were washed thrice with serum-free medium after growing to ~80% confluence. Then, after incubating in a serum-free medium for 24h, the conditioned medium (CM) was collected. 1X10^6^ neutrophils were seeded on 6-well plates with RPMI 1640 medium, adding 10% si-TNFAIP6 or si-NC LUAD cells CM. After 16h at 37°C, the total RNA and protein were collected. The expression of Fas cell surface death receptor (FAS), C-C motif chemokine ligand 3 (CCL3), intercellular adhesion molecule 1 (ICAM-1), C-C motif chemokine ligand 2 (CCL2), C-X-C motif chemokine receptor 4 (CXCR4) and vascular endothelial growth factor A (VEGF-A) were detected.

### 2.11 Western blot

Western blot was performed as previously described (35). Primary antibodies used were: anti-TNFAIP6 (1:1000, Proteintech, China), anti-TLR6 (1:1000, ABclonal, China), anti-FAS (1:3000, Proteintech, China), anti-CCL3 (1:1000, Proteintech, China), anti-ICAM-1 (1:3000, Proteintech, China), anti-CCL2 (1:3000, Proteintech, China), anti-CXCR4 (1:3000, Proteintech, China), anti-VEGFA (1:2000, Proteintech, China), anti-β-Tubulin (1:20000, Proteintech, China) and anti-GAPDH (1:2000, Servicebio, China).

### 2.12 Annexin V- PI assay

The Annexin V- PI assay was performed as previously described (36). Briefly, after being stained with the Annexin V-FITC and PI (BD Biosciences, CA, USA) for 15 mins, the cells were analyzed using the Agilent Novocyte 2000R flow cytometer (Agilent Technologies, USA).

### 2.13 Cell counting Kit-8 assay

CCK8 assay was performed as previously described (31). Briefly, 4000 cells of each cell line were seeded in a 96-well plate. 10μl CCK8 (APExBIO, USA) was added to each well after 24h, 48h, and 72h incubation. Then, the OD values were detected after 1h incubation.

### 2.14 CM collection and protein precipitation

The CM was collected and centrifuged at 1000g for 5 minutes. The supernatant was centrifugated for 30 minutes using the Amicon^®^ Ultra-15 Centrifugal Filter Unit (3 KDa, Millipore, USA). Afterward, the concentrated liquor was mixed with an equal volume of 50% Trichloroacetic acid (TCA) solution and incubated on ice for 2 hours. Then, the protein precipitation was obtained after centrifuged at 2000g and 4°C for 5 minutes and washed twice with 1ml of pre-chilled (-20°C) acetone.

### 2.15 In-solution digestion

The precipitated proteins were suspended in 100 mM NH_4_HCO_3_ and incubated overnight at 37°C with trypsin (Promega, USA). Subsequently, the solutions were heated at 56°C for 1h with 5mM dithiothreitol, followed by alkylation in the dark for 45 min with 15mM iodoacetamide. The unreacted iodoacetamide was then neutralized at room temperature for 30 min with 30mM cysteine. A second trypsin digestion was performed at 37°C for 4h and stopped with 10% TFA. The resulting solutions were dried using SpeedVac and desalted using a μ-C18 Ziptip (Millipore, USA).

### 2.16 Liquid chromatography-tandem mass spectrometry analysis

After desalting, each tryptic digest was dissolved in HPLC buffer A (0.1% (v/v) formic acid in water) and injected into a nano-LC system (EASY-nLC 1200, Thermo Fisher Scientific, USA). Each sample was separated using a C18 column (75μm inner-diameter×25 cm, 3 μm C18) with a 130 min HPLC gradient at a flow rate of 300 nL/min. The gradient consisted of the following steps: 5% to 7% solvent B (0.1% formic acid in 80% acetonitrile) in 2 min, 7% to 22% solvent B in 78 min, 22% to 38% solvent B in 38 min, 38% to 100% solvent B in 3 min, and hold at 100% solvent B for 9 min. The HPLC eluate was directly electrosprayed into an Orbitrap Eclipse Tribrid mass spectrometer (Thermo Fisher Scientific, USA). The spray voltage was set to 2.2 kV, the funnel RF level was set at 40, and the ion transfer tube temperature was set at 320°C. Mass spectrometric analysis was performed in a data-dependent (DDA) mode with a 2s cycle, and data acquisition was carried out using Xcalibur (v.4.5). The orbitrap mass analyzer was utilized as the MS1 detector with a resolution of 60,000 and a scan range of 350–1500 m/z. The normalized AGC target and maximum injection time were set at 100%/50 ms for MS1, and 100%/22 ms for MS2. The orbitrap mass analyzer was employed as the MS2 detector with a resolution of 15000. Precursor ions with charges of +2 to +5 were selected for MS2, and a dynamic exclusion time of 55s was set. The MS2 isolation window was 1.6Da, and precursor fragmentation was achieved using a normalized HCD (higher-energy collision-induced dissociation) collision energy of 30%.

### 2.17 Database search

Proteome Discoverer (PD) 3.0 was used to search the MS/MS data, with a maximum false discovery rate (FDR) of 1% for peptides. Peptide sequences were searched with trypsin specificity, allowing a maximum of two missed cleavages. Fixed modification of carbamidomethylation on cysteine was specified, and the minimal peptide length was set to six. Variable modifications included methionine oxidation and acetylation on the N-terminal and lysine residues. The mass tolerances for precursor ions were set at ±10 ppm and ±0.02 Da for MS/MS.

### 2.18 Label-free quantification

Protein abundance was determined by summing the abundances of unique+trazor peptides, and the PD3.0-derived abundance ratio was used for protein quantitation. The fold difference per protein was calculated from the average abundance (normalized) in all replicates, and the t-test was applied to assess the statistical significance. The abundance was required in at least two replicates for protein quantification to be considered.

### 2.19 Statistical analysis

Statistical analysis was performed by R software (version 3.6.4) or SPSS version 23. T-tests were used for the data with homogeneity of variance. Mann-Whitney U-tests were used for the data without homogeneity of variance. Wilcoxon Rank Sum and Signed Rank Tests were used for unpaired data. Kruskal tests were used for samples with multiple groups. The correlation analysis was performed using Pearson’s correlation coefficient. The survival analysis was tested by Log-rank test. P-value <0.05 was considered significant.

## 3 Results

### 3.1 Screening of the hub genes associated with neutrophil infiltration in LUAD

**Figure 1:**
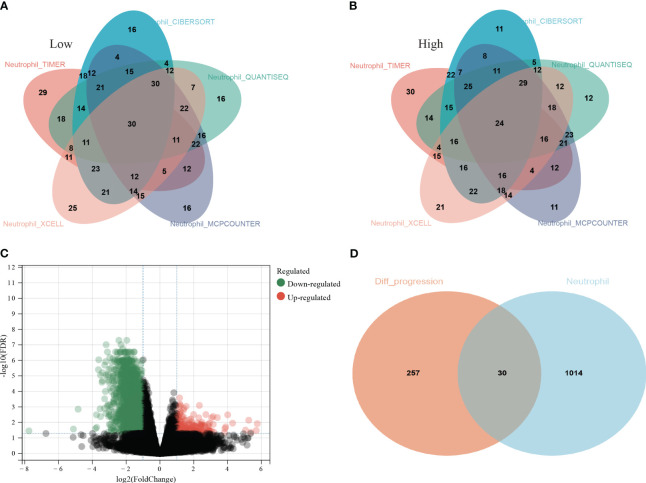
Screening process of the thirty hub genes. **(A)** Thirty lung adenocarcinoma (LUAD) patients from TCGA database were identified as low neutrophil infiltration according to TIMER, CIBERSORT, QUANTISEQ, XCELL, and MCPCOUNTER analysis; **(B)** twenty-four LUAD patients were identified as high neutrophil infiltration; **(C)** The differentially expressed genes (DEGs) between high and low neutrophil infiltration patients; **(D)** 287 genes among the DEGs were associated with PFS (*orange circle*) and 1044 genes specifically elevated in neutrophils were download from THPA (*cyan circle*). Thirty genes were ultimately selected as hub genes associated with neutrophil infiltration in LUAD (*overlap region*).

All LUAD data were extracted from the TCGA database. The neutrophil infiltration in each sample was scored via five independent methods: TIMER, CIBERSORT, QUANTISEQ, XCELL, and MCPCOUNTER. Then, all patients were divided into low and high infiltration groups. Thirty patients with low infiltration (**Figure 1A**) and 24 with high infiltration (**Figure 1B**) were identified in all five methods.

Then, the DEG analysis was performed between the two groups (**Figure 1C**). Among these DEGs, 287 genes were confirmed as significantly associated with PFS. After intersecting these 287 genes and neutrophils-specific elevated genes retrieved from THPA, 30 genes were selected as hub genes ultimately (**Figure 1D**). The official symbols and univariate Cox regression analysis of these 30 hub genes were listed in **Table 1**. The results of other PFS-associated DEGs were shown in **Supplementary Table 3**.

**Table 1:** Official symbols and univariate Cox regression analysis of the 30 hub genes.

Official symbols of the 30 hub genes	Hazard ratio (HR)	HR 0.95L	HR 0.95H	P-value
RNF175	0.794973	0.638474	0.989833	0.040236
CFP	0.813738	0.671622	0.985927	0.035319
SCARF1	0.835366	0.713439	0.97813	0.02544
DPEP2	0.844535	0.717108	0.994604	0.042888
PRAM1	0.849817	0.729501	0.989976	0.03668
NCF1	0.858761	0.73832	0.99885	0.048281
GPX3	0.866249	0.77219	0.971764	0.01435
TLR2	0.870601	0.780534	0.97106	0.012882
P2RY13	0.872838	0.767962	0.992037	0.037308
CYP27A1	0.873393	0.77547	0.983683	0.025673
TMEM130	0.896336	0.818025	0.982143	0.018964
ALPL	0.925434	0.864133	0.991084	0.026685
C4BPA	0.938682	0.886638	0.993782	0.029683
TNFAIP6	1.137979	1.013886	1.27726	0.028231
NAMPT	1.143029	1.01787	1.283578	0.023864
PLAUR	1.161761	1.027196	1.313953	0.016978
SOD2	1.167791	1.003731	1.358666	0.044624
ITGA5	1.169639	1.027687	1.331198	0.017612
RGS2	1.174756	1.043412	1.322634	0.007757
MBOAT2	1.180258	1.023182	1.361448	0.022937
DDX58	1.188232	1.0098	1.398193	0.037761
ITPRIP	1.193036	1.002325	1.420033	0.047023
RELL1	1.195204	1.032019	1.384192	0.017277
MCTP1	1.209421	1.030428	1.419507	0.019978
PCSK5	1.20998	1.021865	1.432725	0.027047
FOSL2	1.22138	1.045147	1.42733	0.011891
MXD1	1.237342	1.056558	1.449059	0.008226
TLR6	1.258264	1.055564	1.499889	0.010367
SLC2A14	1.289774	1.030839	1.613751	0.02604
KIAA0825	1.47937	1.18471	1.847316	0.000549

### 3.2 Consensus clustering analysis based on the 30 hub genes

**Figure 2:**
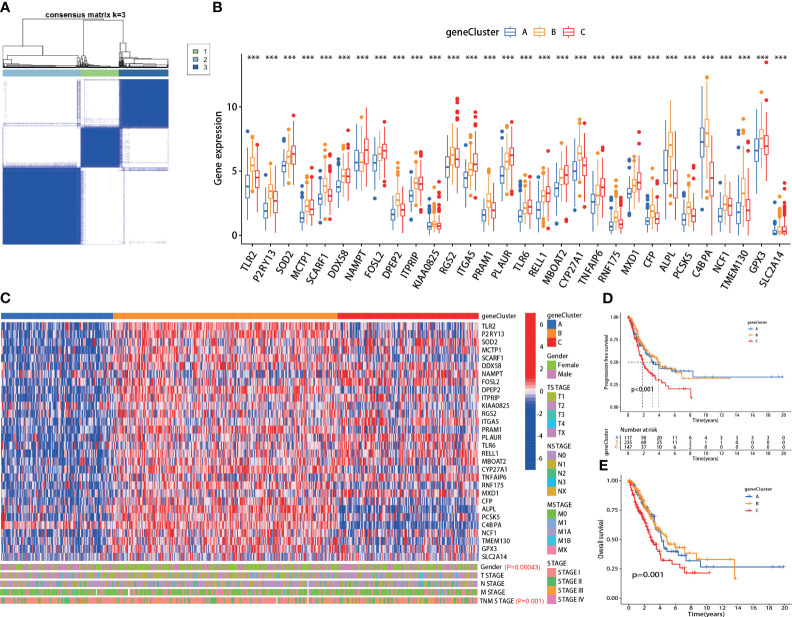
The hub genes correlated with clinical characteristics in lung adenocarcinoma (LUAD). **(A)** All LUAD patients in TCGA were divided into A, B, and C groups by consensus clustering analysis according to the 30 hub genes; **(B)** The expression of each hub gene was significantly different among the three groups; **(C)** *Top panel*: heatmap of the 30 hub genes in each group; *Bottom panel*: each group exhibited different clinical features in gender and TNM staging; **(D, E)** The A, B and C group patients showed significant difference in PFS (p<0.001) and OS (p=0.001). ***:p-value < 0.001.

Using the screened 30 hub genes mentioned above, we performed consensus clustering analysis on all LUAD patients in TCGA, dividing them into three groups - A, B, and C (**Figure 2A**). Each gene showed differential expression among the three groups (**Figure 2B**). Group A mainly exhibited lower expression of the 30 genes, Group B had significantly higher expression, and Group C displayed intermediate levels (**Figure 2C**, *top panel*). The groups also showed significant differences in gender distribution (P=0.00043) and TNM staging (P=0.001) (**Figure 2C**, *bottom panel*). Moreover, significant differences in prognosis were observed, with Group C having the lowest PFS (P<0.001) and OS (P=0.001) (**Figures 2D, E**). Additionally, each group exhibited differential prognostic trends in disease-free survival (DFS) (P=0.072) and significant differences in disease-specific survival (DSS) (P=0.002) (**Supplementary Figures 1A, B**).

### 3.3 Neutrophil infiltration scoring in LUAD

#### 3.3.1 Validation of neutrophil scoring model

**Figure 3:**
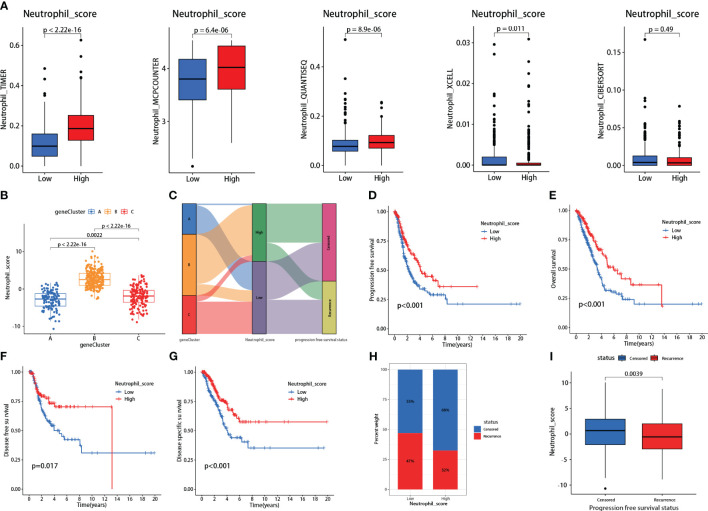
Validations and prognosis predicting effects of the neutrophil scoring model. **(A)** Based on the hub genes, a new neutrophil scoring model was developed by PCA algorithm and validated via five independent tumor-infiltrating immune cells (TICs) analysis methods; **(B)** Each group from consensus clustering analysis exhibited a significant difference in neutrophil sore; **(C)** Group C presented the highest proportion of recurrences; **(D–G)** low neutrophil score patients exhibited significantly lower PFS (P<0.001), OS (P<0.001), DFS (P=0.017), and DSS (P<0.001); **(H)** Low neutrophil score patients presented higher recurrence rate (47% vs 32%); **(I)** Recurrent patients also exhibited lower neutrophil score (P=0.0039).

The neutrophil scoring model was developed using the PCA algorithm depending on the 30 hub genes. All LUAD patients from TCGA were divided into high and low neutrophil score groups. Five independent TICs analysis methods were used to validate the effectiveness of this new scoring model. Results showed that patients with high scores showed significantly increased neutrophil infiltration scores in TIMER, MCPCOUNTER, and QUANTISEQ (P<0.001 for all) (**Figure 3A**). In XCELL, high-score patients exhibited lower neutrophil infiltration (P=0.011) (**Figure 3A**). In CIBERSORT, there was no difference between the two groups (**Figure 3A**).

We further assessed the neutrophil scores in the three patient groups mentioned in the consensus clustering analysis. Results showed that Group A had the lowest scores, followed by Group C, and Group B had the highest scores, with significant differences among the groups (**Figure 3B**). The scores aligned with their gene expression profiles. Meanwhile, consistent with the clustering analysis, Group C had the highest proportion of recurrences (**Figure 3C**).

#### 3.3.2 Neutrophil scoring positively correlated with prognosis

Survival analysis revealed that patients with low neutrophil scores presented significantly lower PFS (P<0.001), OS (P<0.001), DFS (P=0.017), and DSS (P<0.001) (**Figures 3D–G**). The low-scoring group also showed a higher recurrence rate than the high-scoring group (47% vs. 32%) (**Figure 3H**). Following stratified analyses demonstrated that low neutrophil score patients also exhibited significantly lower PFS in aged over 65(P<0.001), female (P=0.006), male (P=0.034), and stages I-II (P=0.006) class (**Supplementary Figure 2**). Neutrophil scores were significantly lower in recurrent patients than in non-recurrent patients (P=0.0039) (**Figure 3I**). Meanwhile, the low neutrophil score group also presented significantly worse OS (P=0.0038) (**Supplementary Figure 3**) in data derived from GSE50081.

#### 3.3.3 Neutrophil scoring correlated with PD-L1 and TMB and promoted their prognosis-predicting capability

**Figure 4:**
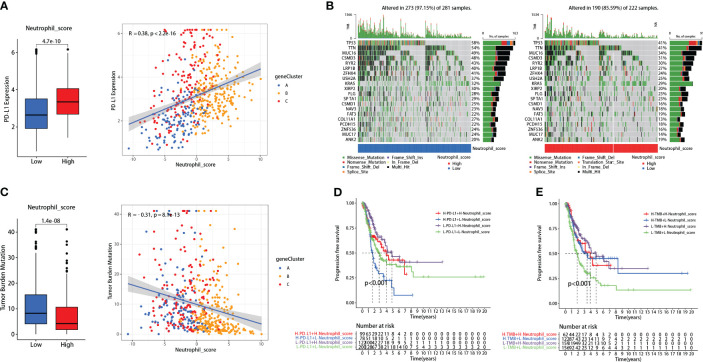
Neutrophil score correlated with PD-L1 and tumor mutational burden (TMB). **(A)** Neutrophil score positively correlated with PD-L1; **(B)** 273 of 281 (97.15%) low-score patients and 190 of 222 (85.59%) high-score patients exhibited gene mutations; **(C)** Neutrophil score negatively correlated with TMB; **(D)** Neutrophil score significantly promoted the FPS predicting effectiveness of PD-L1; **(E)** It also promoted the effectiveness of TMB.

Patients with high neutrophil sore presented higher PD-L1 expression (P<0.001) (**Figure 4A**). Further correlation analysis demonstrated that neutrophil score was significantly positively correlated with PD-L1 expression in LUADs (R=0.38, P<0.001) (**Figure 4A**). TMB, on the contrary, presented significant reverse results as negatively correlated with neutrophil score (R=-0.31, P<0.001) (**Figure 4C**). The genetic mutation status of the two groups was also basically consistent with TMB, as 273 of 281 (97.15%) in the low-score group and 190 of 222 (85.59%) in the high-score group exhibited gene mutations (**Figure 4B**).

Interestingly, the neutrophil scoring might improve the prognosis-predicting capability of PD-L1 and TMB in lung adenocarcinoma. Patients with high PD-L1 expression had slightly worse PFS than those with low PD-L1 expression (P=0.039), while there was no significant difference between high and low TMB patients (P=0.139) (**Supplementary Figure 4**). When combined with neutrophil scoring, respectively, significant differences were observed among each group in both PD-L1 (P<0.001) (**Figure 4D**) and TMB (P<0.001) (**Figure 4E**).

#### 3.3.4 Neutrophil scoring correlated with tumor immune microenvironment (TIME) in lung adenocarcinoma

**Figure 5:**
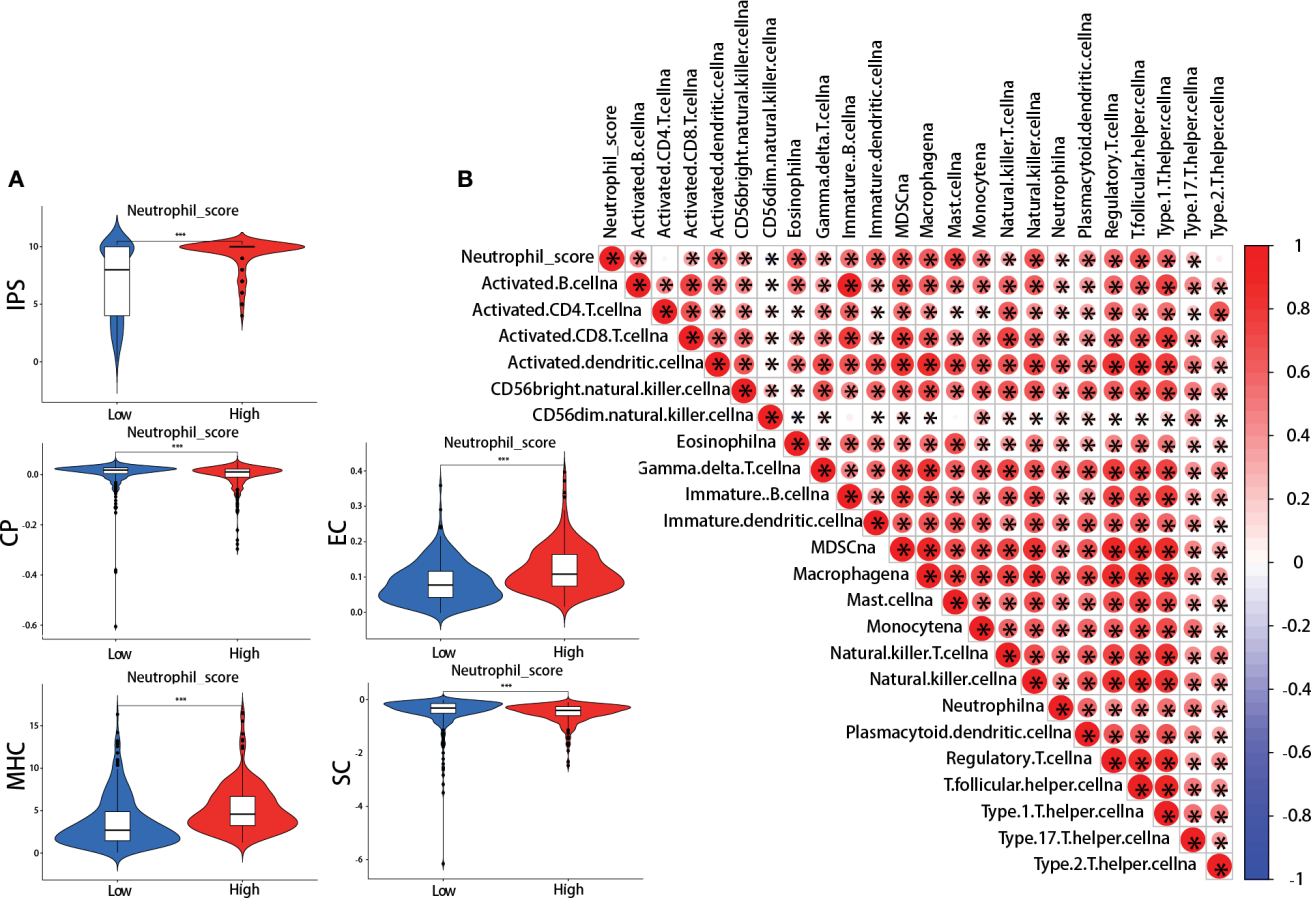
Neutrophil score and tumor immune microenvironment (TIME). **(A)** ImmunoPhenoScore (IPS): the high-scoring group presented higher total IPS, effector cells (EC), and major histocompatibility complex (MHC) scores, and lower checkpoints (CP) and suppressor cells (SC) scores; **(B)** The correlations between neutrophil score and other cells in TIME. *: p-value < 0.05; ***:p-value < 0.001.

IPS was performed between the high and low neutrophil score groups. We found that the high-scoring group presented significantly higher effector cells (EC) and major histocompatibility complex (MHC) scores and lower checkpoints (CP) and suppressor cells (SC) scores (**Figure 5A**). The IPS total score was also higher in the high-scoring group (**Figure 5A**). Meanwhile, further analysis revealed that neutrophil score was closely related to the infiltration of other immune cells, including cytotoxic CD8 + T cells, effector CD4 + T cells, natural killer cells (NK), Tregs, myeloid-derived suppressor cells (MDSCs), and macrophages (**Figure 5B**). The neutrophil scoring was broadly correlated with TIME in lung adenocarcinoma.

### 3.4 Ten genes, including TNFAIP6, might be correlated with the pro-tumor effects of TANs

**Figure 6:**
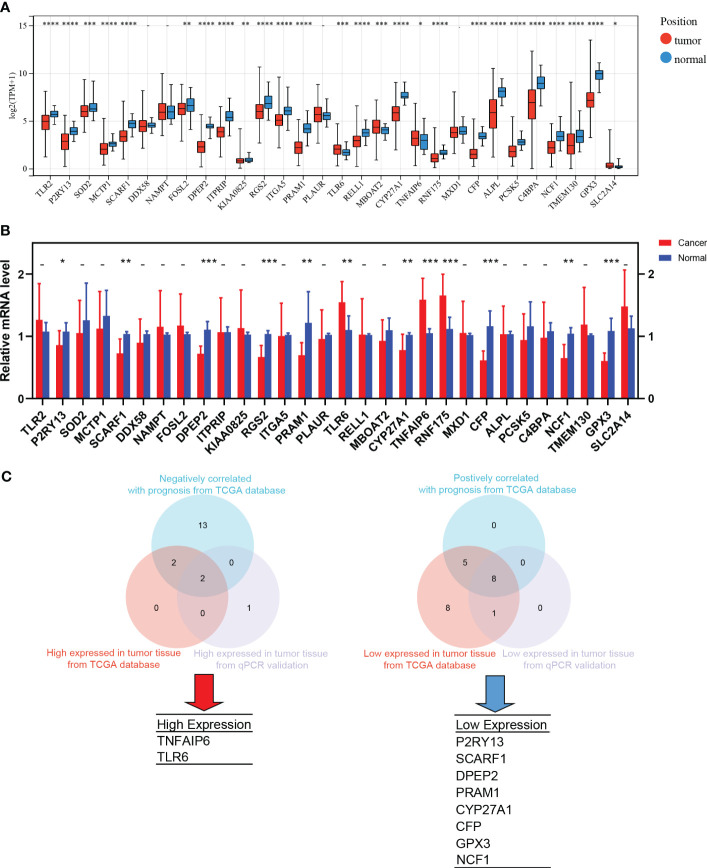
The screening process of pro-tumor effects associated genes. **(A)** Differential expression of the 30 hub genes between tumor and normal tissues in LUAD from TCGA; **(B)** The differential expression was further validated in the fresh samples collected from our department; **(C)** The strategy and results of the pro-tumor effects associated genes screening. *: p-value < 0.05; **: p-value < 0.01; ***:p-value < 0.001; ****: p-value < 0.0001.

TANs exhibit both pro-tumor and anti-tumor effects in the tumor microenvironment. We performed the following procedures to screen for the genes closely associated with the pro-tumor effects of TANs. Firstly, we analyzed the differential expression of the 30 hub genes between tumor and normal tissues in LUAD patients from the TCGA database (**Figure 6A**). We also collected paired cancer and normal tissues from our department’s surgical resections of LUAD patients. The differential expression of these 30 hub genes was further validated in those fresh specimens via qPCR (**Figure 6B**). The hazard ratio of each hub gene has been assessed via univariate Cox regression, as mentioned above (**Table 1**).

After taking the intersection, we found that TNFAIP6 and TLR6 were not only overexpressed in cancer tissue but also indicated poorer prognosis (**Figure 6C**). The P2RY13, SCARF1, DPEP2, PRAM1, CYP27A1, CFP, GPX3, and NCF1 were low expressed in cancer tissue and indicated better prognosis (**Figure 6C**). We speculated that dysregulation of these ten genes might indicate the pro-tumor effects of TANs in lung adenocarcinoma.

### 3.5 TNFAIP6 overexpressed in lung adenocarcinoma cells and might promote neutrophil “N2” polarization *in vitro*

**Figure 7:**
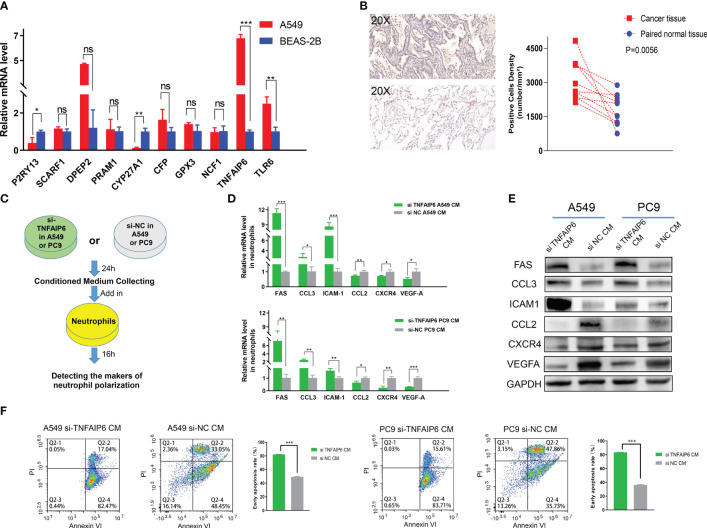
TNFAIP6 and pro-tumor effects. **(A)** TNFAIP6, TLR6, P2RY13, and CYP27A1 were significantly dysregulated in all A549, PC9, and H1975 cells (results of PC9 and H1975 shown in **Supplementary Figure 5**), compared to BEAS-2B cells; **(B)** Immunohistochemical (IHC) staining of TNFAIP6. *Left panel*: typical IHC staining images of lung adenocarcinoma (LUAD) tissue (*top part*) and normal tissue (*bottom part*); *Right panel*: statistical analysis showed TNFAIP6 significantly overexpressed in LUAD tissues; **(C)** Flowchart of the co-culturing procedure; **(D, E)** Knocking down TNFAIP6 in A549 and PC9 resulted in upregulating of FAS, CCL3, and ICAM-1 and downregulating of CCL2, CXCR4, and VEGF-A in neutrophils; **(F)** Knocking down TNFAIP6 in A549 (*left panel*, P<0.001) and PC9 (*right panel*, P<0.001) significantly evaluated the early apoptosis rate of neutrophils. ns: p-value≥0.05; *: p-value<0.05; **: p-value < 0.01; ***:p-value < 0.001.

In order to further screen the potential genes differentially expressed in tumor cells specifically, instead of TICs in TIME, we used qPCR to test the expression of all the ten genes in the A549, PC9, and H1975 cell lines with the BEAS-2B cell line as a control. The results showed that TNFAIP6, TLR6, P2RY13, and CYP27A1 were significantly differentially expressed in all A549, PC9, and H1975 cell lines (**Figure 7A**; **Supplementary Figure 5**). The TNFAIP6 protein was significantly overexpressed in cancer tissue compared to normal pulmonary tissue, according to the IHC examination (**Figure 7B**). Then, the TNFAIP6 was knocked down in both A549 and PC9 cells (**Supplementary Figure 6**). The CCK8 assay showed that TNFAIP6 might not affect the proliferation of A549 and PC9 cells (**Supplementary Figure 7**). After co-culturing healthy human neutrophils with the conditioned medium (**Figure 7C**), we found that knocking down TNFAIP6 in A549 and PC9 led to the elevated expression of FAS, CCL3, and ICAM-1 in neutrophils while reducing the expression of CCL2, CXCR4, and VEGF-A (**Figures 7D, E**). The CM derived from TNFAIP6 knock-downed A549 and PC9 cells also promoted the early apoptosis rate of neutrophils (**Figure 7F**). Furthermore, the LC-MS/MS analysis showed that eight cytokines, including glucose-6-phosphate isomerase (GPI), C-X-C motif chemokine ligand 5 (CXCL5), macrophage migration inhibitory factor (MIF), secreted phosphoprotein 1 (SPP1), C-X-C motif chemokine ligand 1 (CXCL1), colony stimulating factor 1 (CSF1), transforming growth factor beta 2 (TGFB2), and CCL2, were secreted into the CMs. The label-free quantification showed that the secretion of SPP1 (P=0.012) and CCL2 (P<0.001) was significantly decreased in si TNFAIP6 CM (**Table 2**).

**Table 2:** Label-free quantification of the differentially secreted cytokines between si NC and si TNFAIP6 A549 cells.

Cytokines	Abundance Ratio: (si NC)/(si TNFAIP6)	Abundance Ratio Adj. P-Value	Abundance Ratio Variability [%]	Score Sequest HT:
GPI	0.698	0.844103815	40.99	1682.79
CXCL5	0.531	0.499366075	58.51	1276.87
MIF	0.555	0.57501736	39.93	396.26
SPP1*	2.737	0.012491252	65.39	391.73
CXCL1	1.058	0.872092929	66.63	239.71
CSF1	0.523	0.479401422	17.95	202.3
TGFB2	1.394	0.555152029	21.94	136.31
CCL2***	4.485	4.62414E-05	17.6	63.43

PS: * 0.05>P>0.01, ***P<0.001.

## 4 Discussion

The effects of TANs on lung adenocarcinoma remain unclear (22, 23). The specific gene sets that can assess neutrophil infiltration are still uncertain. In this study, we employed the five most widely used TICs analysis methods, based on the TCGA database, to explore the DEGs between high and low neutrophil infiltration patients. Then, by intersecting DEGs, prognosis effects, and neutrophils-specific expressed genes from THPA, we preliminarily identified 30 hub genes (**Figure 1**; gene symbols were listed in **Table 1**). Then, subsequent consensus clustering analysis validated that these hub genes might be widely associated with clinical pathological features and prognosis in LUAD (**Figure 2**; **Supplementary Figure 1**).

The specific neutrophil infiltration scoring in LUAD has not been reported yet. Thus, based on these 30 hub genes, we developed a comprehensive scoring system that might be able to evaluate the neutrophil infiltration in LUADs precisely. The following multiple-method validation revealed that this scoring system effectively reflected neutrophil infiltration status and correlated with essential clinical characteristics (**Figures 3A–C**).

Due to the dual effects of neutrophils on cancers, the prognosis prediction in LUAD by traditional neutrophil infiltration scoring methods was ambiguous. For example, *Xinyan Liu* et al. reported no significant association between neutrophil infiltration and prognosis in LUAD (23). In contrast, *Mehrdad Rakaee* et al. reported that high neutrophil infiltration density suggests a poor prognosis in LUAD (22). Unlike other TICs analysis methods, our neutrophil infiltration scoring system was developed based on the prognosis-related hub genes. It was significantly associated with PFS, OS, DFS, DSS, and recurrence rate in LUADs (**Figures 3D–I**). Meanwhile, we further validated the prognosis predictive capability of the scoring system in an independent cohort, resulting in similar outcomes (**Supplementary Figure 3**).

Our neutrophil scoring system might apply to immune checkpoint inhibitors (ICIs) efficacy prediction. Over the past decade, research outcomes regarding ICIs have revolutionized the lung cancer treatment landscape (37). For instance, Pembrolizumab has significantly improved the 5-year survival rate of advanced NSCLC patients and has been approved for first-line treatment in patients with PD-L1 tumor proportion score (TPS) ≥1% and without EGFR/ALK gene alterations (38, 39). Recent studies have underscored the pivotal role of TANs in the anti-tumor immune response, showing their capacity to disrupt ICI responses and their correlation with ICIs acquired resistance (40). Our results showed that the neutrophil score positively correlated with PD-L1 expression and negatively correlated with TMB (**Figures 4A, C**). PD-L1 and TMB are vital biomarkers for predicting ICI efficacy (41, 42). Our neutrophil infiltration scoring system maintained a significant association with PD-L1 and TMB and presented independence from these two biomarkers. These results indicated the potential predictive role of neutrophil sore in ICI treatment efficacy.

Meanwhile, the prognostic significance of PD-L1 and TMB in LUAD remains uncertain. Although the overexpressed PD-L1 was reported to be significantly associated with poor prognosis (43), the predictive effect of TMB on prognosis is less robust (44, 45). Consistent with these researches, our data also exhibited similar limitations of PD-L1 and TMB (**Supplementary Figure 4**). As the neutrophil soring might be independent of both PD-L1 and TMB, as discussed above, we combined these effects. Our neutrophil soring significantly improved the prognostic prediction of PD-L1 and TMB, respectively (**Figures 4D, E**).

The potential predictive value of neutrophil score for ICI efficacy and prognosis of LUAD patients might be attributed to the broad crosstalk between TANs and other cells in TIME (46). The TIME mainly comprises CD8 + T cells, CD4 + T cells, NK, Tregs, MDSCs, macrophages, etc (47). These cells can influence neutrophil infiltration and function by secreting chemokines to recruit neutrophils, inducing “N2” polarization, etc (1, 48). The TANs, on the other hand, also affect other cells in TIME, such as forming NETs to diminish the cytotoxic effects of CD8+ T cells on tumor cells (49). Our results indicated similar crosstalk effects on the bioinformatics level, as the neutrophil score was significantly associated with the IPS score and other TICs in LUAD (**Figure 5**).

Based on neutrophil sore potential values, we believed the 30 hub genes might be strongly associated with the infiltration of neutrophils in LUAD. They might extensively participate in the mechanisms of the neutrophil effect on cancer cells and TIME. As mentioned earlier, TANs exhibit a significant dual role in promoting and inhibiting tumor growth, named pro-tumor and anti-tumor effects. To further explore which specific genes are associated with TANs’ pro-tumor effects, we integrated bioinformatics analyses, tissue validation, and prognosis analysis in the following investigations.

The results showed that the high expression of TNFAIP6 and TLR6, as well as the low expression of P2RY13, SCARF1, DPEP2, PRAM1, CYP27A1, CFP, GPX3, and NCF1 might be closely associated with pro-tumor effects (**Figure 6**). Thus, we speculated that these genes might be likely crucial regulators or key downstream targets in TANs’ tumor-promoting activities.

Lung cancer tissue comprises tumor cells, extracellular matrix, immune cells, etc. The dysregulation of these ten genes might manifest in various cell types within the tumor tissue. Therefore, to preliminary identify genes that potentially have specific differential expression in tumor cells, we detected the expression of these ten genes in adenocarcinoma cell lines, including A549, PC9, and H1975. The BEAS-2B cell lines, which were isolated from normal human bronchial epithelium, were selected as the control group. Significant upregulation of TNFAIP6 and TLR6 and downregulation of P2RY13 and CYP27A1 were observed in all three adenocarcinoma cell lines (**Figure 7A**; **Supplementary Figure 5**).

TNFAIP6, also known as TSG-6 (50), exhibited anti-inflammatory effects in myocardial infarction and trauma repair (51). The role of TNFAIP6 in tumors was rarely reported. Several studies showed that TNFAIP6 can promote metastasis in gastric and colorectal cancers (52, 53). Its elevated expression has also been significantly associated with poor prognosis in urothelial carcinomas (54). The effect of TNFAIP6 on lung cancer and its TIME has not been reported yet. We knocked down TNFAIP6 expression in lung adenocarcinoma cells by siRNA (**Supplementary Figure 6**) and co-cultured neutrophils within its conditioned medium (**Figure 7C**). The results showed that FAS, CCL3, and ICAM-1 were significantly upregulated, and CCL2, CXCR4, and VEGF-A were downregulated in neutrophils (**Figures 7D, E**).

Various studies have employed FAS, CCL3, ICAM-1, CCL2, CXCR4, and VEGF-A as biomarkers to characterize “N1” and “N2” neutrophil phenotypes (55, 56). Among these, FAS, also known as CD95, is a transmembrane protein that triggers the apoptosis signaling pathway upon binding with FASL (57). *Z.G. Fridlender* et al. revealed that FAS was significantly overexpressed in “N1” polarized neutrophils (48). ICAM-1, an intercellular adhesion molecule that plays a pivotal role in inflammation, was also significantly elevated in “N1” polarized neutrophils, according to investigations conducted by *Mareike Ohms* et al. *in vitro* (58, 59).

CCL3, also known as macrophage inflammatory protein-1α (MIP-1α), might play dual effects in TIME (60). On one hand, its chemotactic function on dendritic cells and CD8+ T cells significantly promoted the anti-tumor effects of immune cells (61). On the other hand, CCL3 also recruited Tregs and MDSCs within the TIME to facilitate immune evasion (62). However, high expression of CCL3 was usually recognized as “N1” polarization in neutrophils (55, 56, 63).

Conversely, overexpressed CXCR4 might indicate “N2” polarization in neutrophils. *Chenghui Yang* et al. reported that aged neutrophils, characterized by high CXCR4 expression, promoted NETs formation, contributing to breast cancer lung metastasis (64). VEGFA, one of the VEGF family proteins, is primarily secreted by neutrophils (65). By binding to VEGFR2 and mediating multiple signaling pathways, VEGFA stimulates angiogenesis and promotes cancer progression in multiple cancers (66). Therefore, elevated VEGFA in neutrophils usually indicates “N2” polarization.

CCL2, known as monocyte chemoattractant protein-1 (MCP-1), primarily functions in monocyte chemotaxis (67). Although CCL2 was once considered to stimulate host anti-tumor responses in a T-lymphocyte-independent manner, it was recently widely recognized for its significant pro-tumor effects (68). Patients overexpressing CCL2 in cancer presented a worse prognosis (69). It also promoted proliferation and enhanced stemness in cancer cells (70, 71). *Shao-Lai Zhou* et al. reported that TANs secreted CCL2 to recruit macrophages and Tregs, promoting hepatocellular carcinoma proliferation (72). Overexpressed CCL2 in neutrophils might promote its pro-tumor effects.

Thus, according to the above literature reports and our experimental results, we speculated that TNFAIP6 overexpressed in lung cancer might induce the “N2” polarization and pro-tumor effects on neutrophils. The “N2” polarization of neutrophils also exhibited a lower apoptosis rate in TIME (63, 73). Our results showed that the neutrophil’s early apoptosis rate was significantly evaluated when treated with si TNFAIP6 CM, further validating our speculation (**Figure 7F**).

There are several limitations in this study. Firstly, we identified the 30 hub genes closely associated with neutrophil infiltration in LUAD and developed a scoring system correlating with prognosis. However, the scoring system solely assessed the infiltration of neutrophils. It was unable to distinguish whether TANs exhibited anti-tumor or pro-tumor effects. The relationship between neutrophil infiltration and prognosis is intricate. Solely evaluating the infiltration might lead to unexpected outcomes in some patients. For instance, in our study, patients in Cluster A, despite having lower scores than Cluster C (**Figure 3B**), exhibited better PFS and OS outcomes (**Figures 2D, E**). Therefore, we further identified 10 out of these 30 hub genes potentially associated with the pro-tumor function of TANs preliminarily. Although the results might be subject to bias, further multi-omic, high-throughput, and multidimensional studies based on these 10 genes could potentially explore evaluation methods capable of simultaneously reflecting TAN infiltration and function.

Meanwhile, our *in vitro* experiments revealed significant upregulation of TNFAIP6 in lung adenocarcinoma cells, which could potentially lead to the “N2” polarization of neutrophils. However, the underlying mechanisms remain unclear. Hence, we performed LC-MS/MS analysis in the CMs. By LFQ, we found that TNFAIP6 significantly stimulated the secretion of CCL2 and SPP1 in LUAD cells (**Table 2**). As discussed above, CCL2 might promote neutrophil “N2” polarization in TIME. SPP1, also known as osteopontin (OPN), is secreted by various cells and plays a crucial role in immune regulation (74, 75). Patients with overexpressed SPP1 in lung cancer presented a poor prognosis (76). The SPP1 also stimulated NETs formation to promote cancer progression (77). Thus, the effects of TNFAIP6 might be attributed to CCL2 and SPP1 secretion. The specific regulatory mechanism requires further investigation. Furthermore, we detected the effects of TNFAIP6 on cancer cell viability by CCK8 assays. Results showed that TNFAIP6 might not or slightly promote LUAD cell proliferation (**Supplementary Figure 7**). Whether TNFAIP6 directly affects cancer cell proliferation also needs further validation.

In addition, whether and how differential expressing TLR6, P2RY13, and CYP27A1 in LUAD cells promote neutrophils’ pro-tumors effects still requires further investigation. Neutrophils contribute to tumor progression through various mechanisms, including N2 polarization, NETs formation, inhibition of NK and CD8+ T cell cytotoxicity, and secretion of pro-angiogenic cytokines (1). Our results in this study indicated that TNFAIP6 might stimulate the neutrophils’ pro-tumor effects by inducing “N2” polarization. However, whether TLR6, P2RY13, and CYP27A1 operate through similar mechanisms remains unclear. For instance, no significant alteration in neutrophil polarization was observed when co-cultured with the CM derived from TLR6 knocking down LUAD cells (**Supplementary Figure 8**). Further comprehensive studies may reveal the underlying mechanisms.

In conclusion, TANs play a crucial role in lung adenocarcinoma. Thirty hub genes identified in this study might broadly participate in the neutrophil effects on LUADs. The neutrophil scoring system, developed based on these 30 hub genes, could effectively predict prognosis and potentially reflect the ICI efficacy and TIME situations in LUADs. 10 of 30 hub genes were further screened as significantly associated with pro-tumor effects of TANs. TNFAIP6, as one of these pro-tumor genes, was significantly overexpressed in lung adenocarcinoma cells and might lead to the “N2” polarization of neutrophils *in vitro*. Further research on these hub genes, provided in this pilot study, may unravel the mechanisms of TANs affecting the TIME and development of LUADs.

## Data availability statement

The raw data supporting the conclusions of this article will be made available by the authors, without undue reservation.

## Ethics statement

The studies involving humans were approved by Ethical Committee of Tianjin Medical University General Hospital. The studies were conducted in accordance with the local legislation and institutional requirements. The participants provided their written informed consent to participate in this study.

## Author contributions

RL: Conceptualization, Funding acquisition, Investigation, Methodology, Visualization, Writing – original draft, Writing – review & editing, Data curation, Project administration, Software, Validation. GZ: Conceptualization, Data curation, Formal analysis, Investigation, Methodology, Software, Validation, Visualization, Writing – review & editing, Writing – original draft. YS: Conceptualization, Data curation, Investigation, Methodology, Software, Validation, Visualization, Writing – original draft, Writing – review & editing. ML: Conceptualization, Investigation, Methodology, Validation, Visualization, Writing – review & editing. ZH: Investigation, Software, Visualization, Writing – review & editing. PC: Conceptualization, Data curation, Writing – review & editing. XL: Conceptualization, Investigation, Methodology, Writing – review & editing. ZS: Conceptualization, Investigation, Validation, Writing – review & editing. JC: Conceptualization, Funding acquisition, Methodology, Writing – review & editing.

## Supplementary material

Supplementary Figure 1 DFS and DSS of the patients grouped by consensus clustering analysis. Lung adenocarcinoma patients were grouped by consensus clustering analysis based on the 30 hub genes. Each group exhibited differential prognostic trends in DFS (P=0.072) **(A)** and significant differences in DSS (P=0.002) **(B)**.

Supplementary Figure 2 Stratified analyses in PFS of the patients with different neutrophil scores. **(A)** No significant difference in PFS was found in the patients aged less than 65 (P=0.098); **(B–E)** Low neutrophil score patients exhibited significantly lower PFS in aged over 65 (P<0.001), female (P=0.006), male (P=0.034), and stages I-II (P=0.006) class; **(F)** For stages III-IV patients, low neutrophil score exhibited lower PFS tendency (P=0.229).

Supplementary Figure 3 Survival analysis of neutrophil score in data from GSE50081. **(A)** The overall survival analysis between low and high neutrophil score groups; **(B)** The receiver operating characteristic curve of the neutrophil score.

Supplementary Figure 4 Survival analysis of PD-L1 and TMB in lung adenocarcinoma (LUAD). **(A)** Lower PD-L1 expression in LUAD patients presented better PFS (P=0.039); **(B)** No significant differences were found between low and high TMB patients.

Supplementary Figure 5 The expression of all the ten genes in PC9 and H1975 cells, compared to BEAS-2B cells. The TNFAIP6, TLR6, P2RY13, and CYP27A1 were significantly differently expressed in both PC9 **(A)** and H1975 **(B)** cells.

Supplementary Figure 6 Knocking down TNFAIP6 in A549 and PC9. **(A)** Si TNFAIP6-2 has strongly inhibited the mRNA expression in A549 cells; **(B)** Si TNFAIP6-2 was selected to transfect into A549 and PC9 and validated by WB.

Supplementary Figure 7 CCK8 assay. TNFAIP6 might not affect the proliferation of A549 **(A)** and PC9 **(B)** cells.

Supplementary Figure 8 TLR6 in lung adenocarcinoma cells might not affect the polarization of neutrophils. **(A)** Si TLR6-1 strongly inhibited the mRNA expression in A549 cells; **(B)** Si TLR6-1 was selected to transfect into A549 and PC9 and validated by WB; **(C)** Knocking down TLR6 in A549 did not affect the expression of FAS, CCL3, ICAM-1, CCL2, CXCR4, and VEGF-A in neutrophils; **(D)** Although knocking down TLR6 in PC9 unregulated the expression of CCL3 (indicating “N1” polarization), it also elevated the expression of CCL2 (indicating “N2” polarization) in neutrophils. Meanwhile, no significant difference was observed in the expression of FAS, ICAM-1, CXCR4, and VEGF-A.
