# Supplementary figures and images for: Neutrophil infiltration associated genes on the prognosis and tumor immune microenvironment of lung adenocarcinoma
Source: Front Immunol. 2023 Dec 22;14:1304529. doi: 10.3389/fimmu.2023.1304529 (PMC10777728; doi:10.3389/fimmu.2023.1304529)

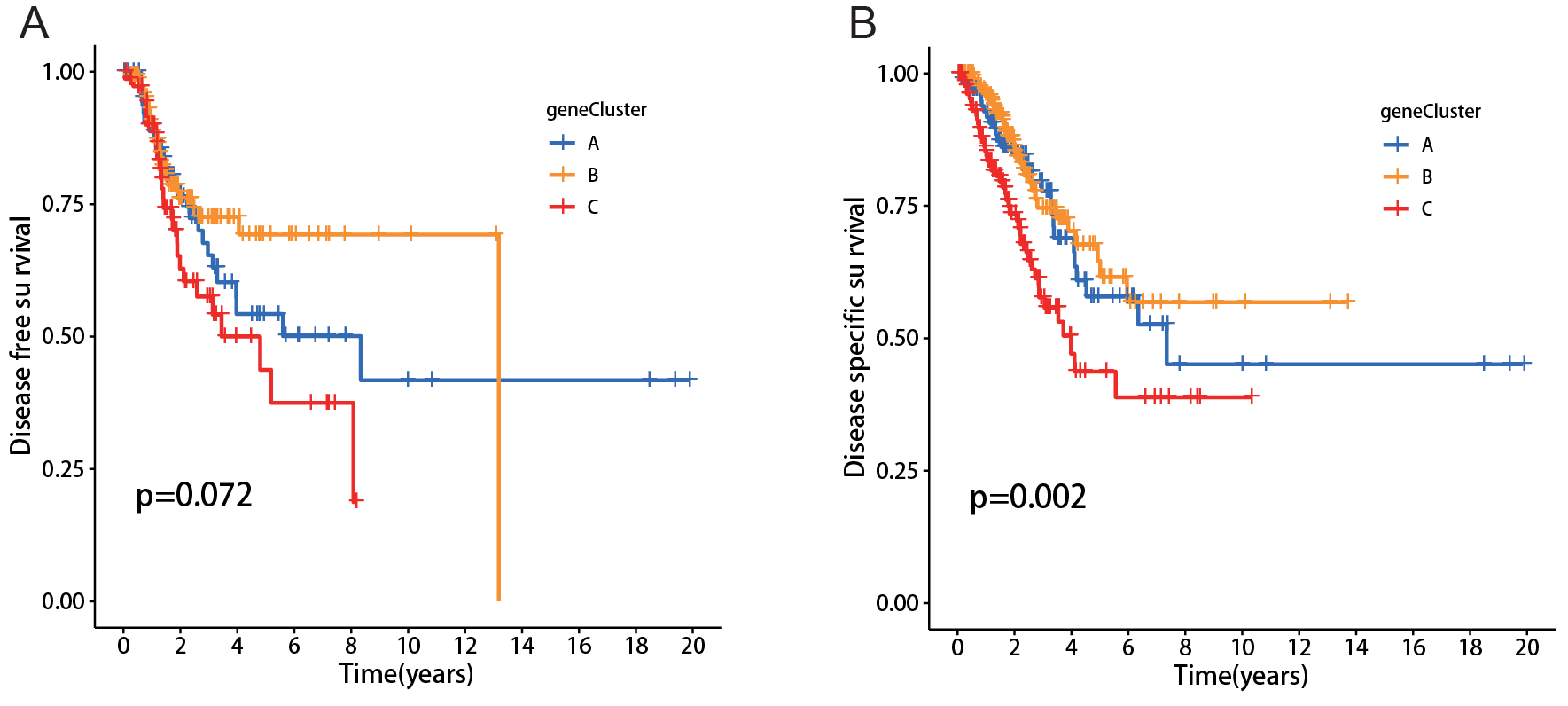

Supplement: Supplementary Figure 1 — DFS and DSS of the patients grouped by consensus clustering analysis. Lung adenocarcinoma patients were grouped by consensus clustering analysis based on the 30 hub genes. Each group exhibited differential prognostic trends in DFS (P=0.072) (A) and significant differences in DSS (P=0.002) (B). [file Image_1.tif]

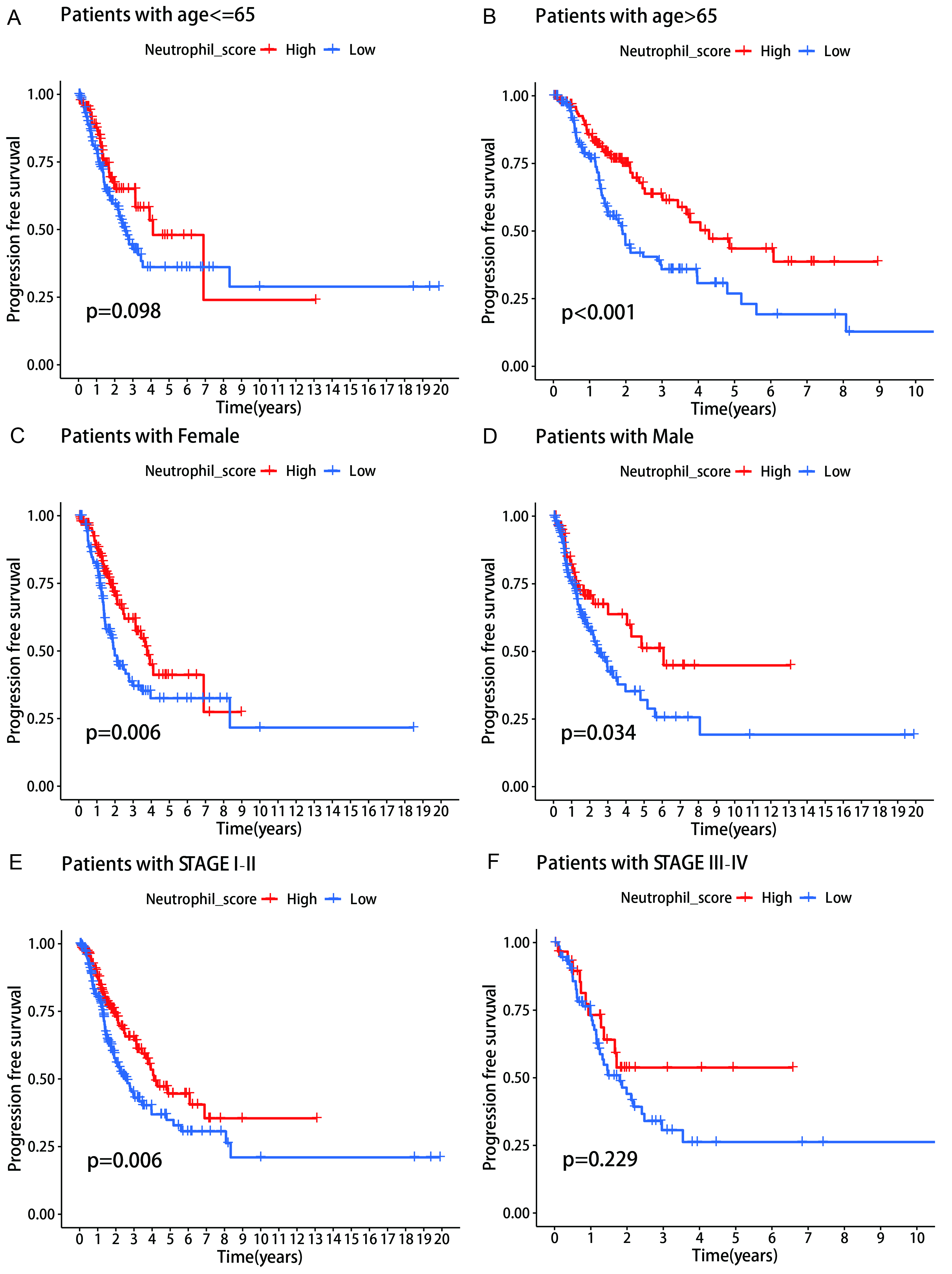

Supplement: Supplementary Figure 2 — Stratified analyses in PFS of the patients with different neutrophil scores. (A) No significant difference in PFS was found in the patients aged less than 65 (P=0.098); (B–E) Low neutrophil score patients exhibited significantly lower PFS in aged over 65 (P<0.001), female (P=0.006), male (P=0.034), and stages I-II (P=0.006) class; (F) For stages III-IV patients, low neutrophil score exhibited lower PFS tendency (P=0.229). [file Image_2.tif]

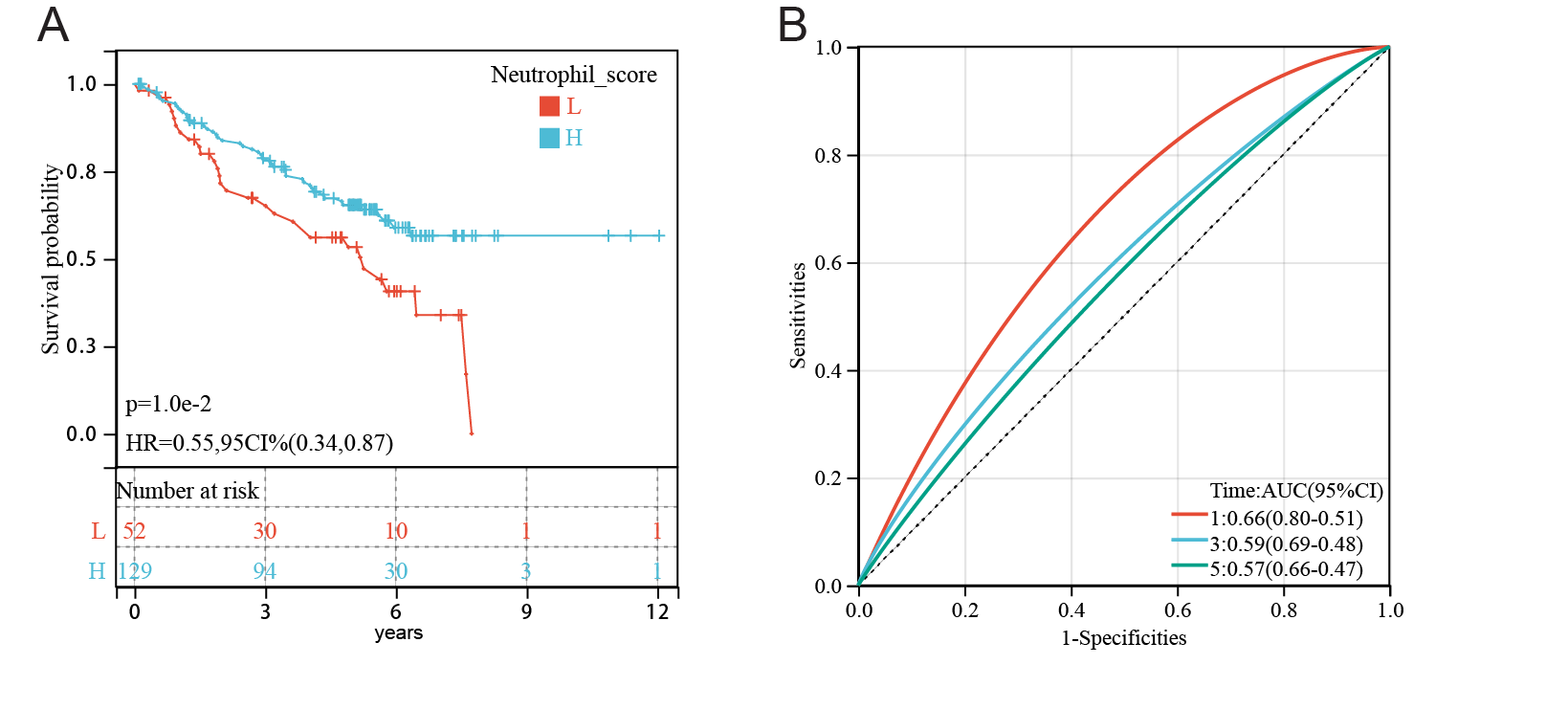

Supplement: Supplementary Figure 3 — Survival analysis of neutrophil score in data from GSE50081. (A) The overall survival analysis between low and high neutrophil score groups; (B) The receiver operating characteristic curve of the neutrophil score. [file Image_3.tif]

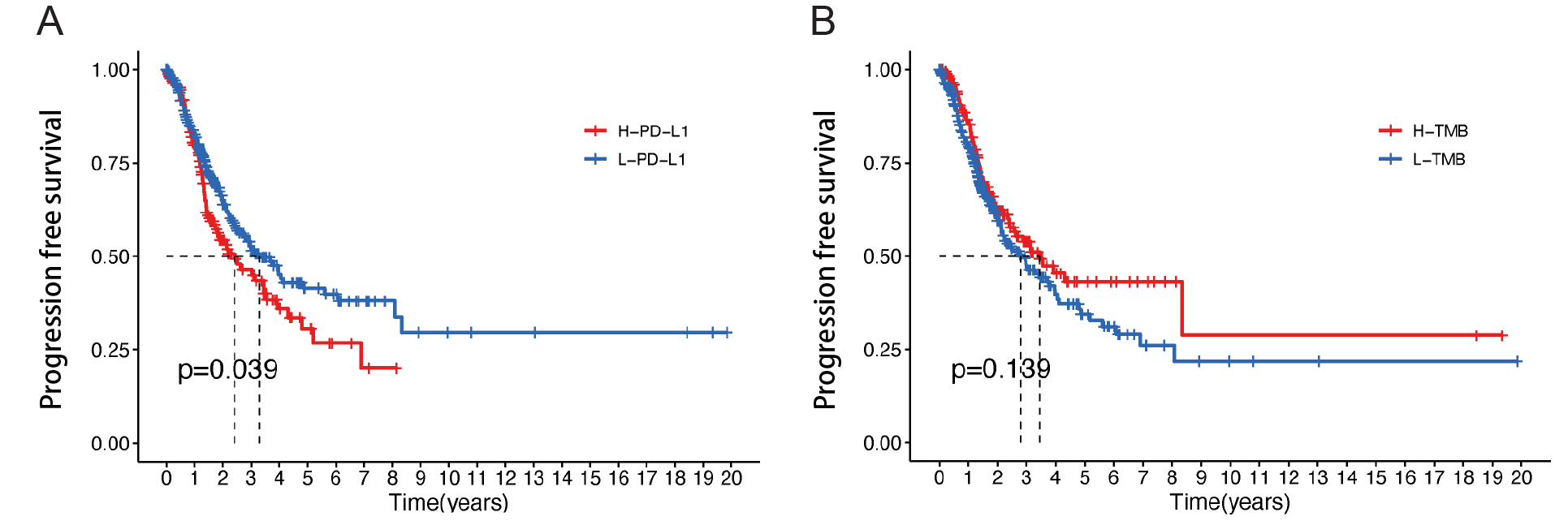

Supplement: Supplementary Figure 4 — Survival analysis of PD-L1 and TMB in lung adenocarcinoma (LUAD). (A) Lower PD-L1 expression in LUAD patients presented better PFS (P=0.039); (B) No significant differences were found between low and high TMB patients. [file Image_4.tif]

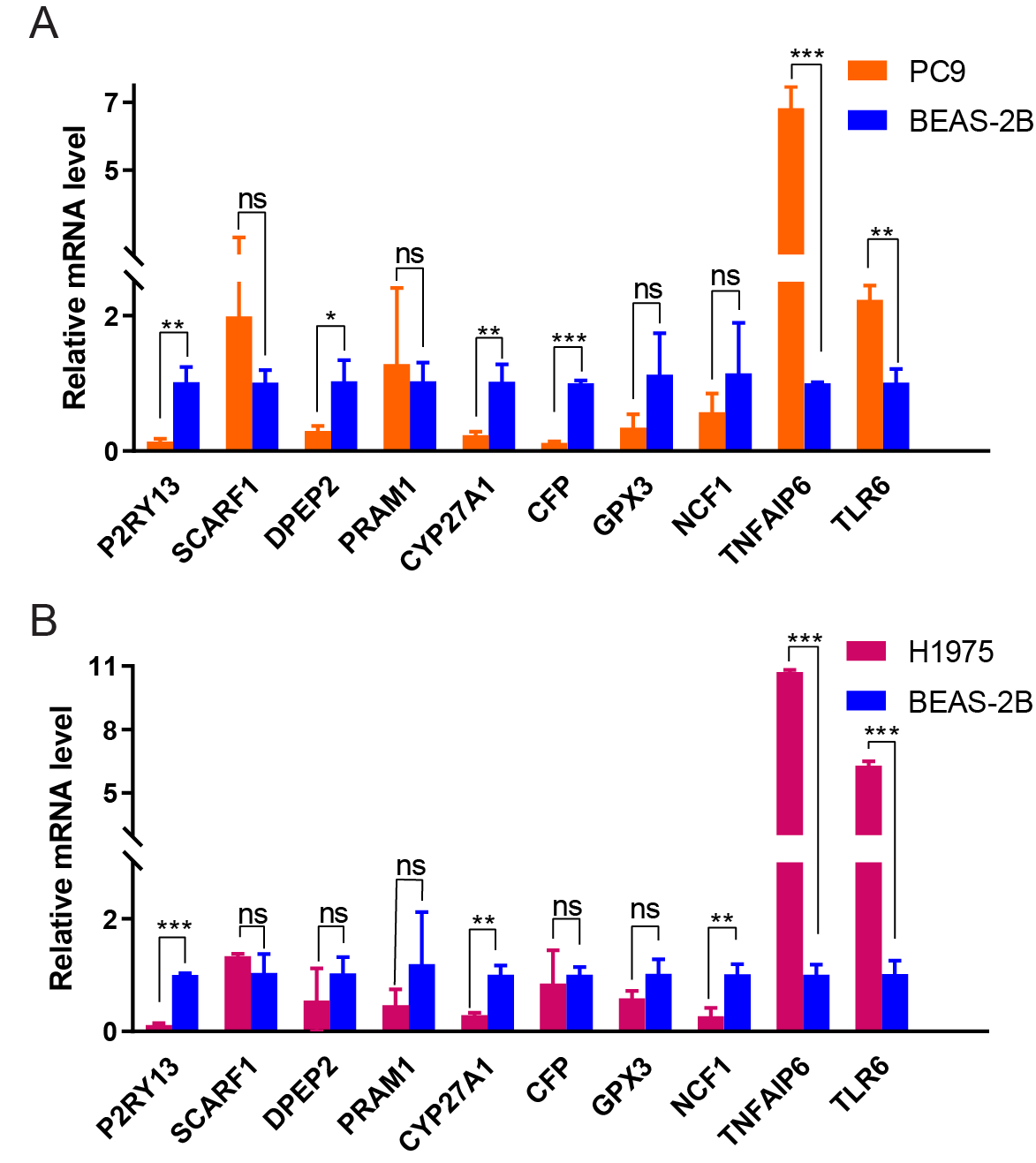

Supplement: Supplementary Figure 5 — The expression of all the ten genes in PC9 and H1975 cells, compared to BEAS-2B cells. The TNFAIP6, TLR6, P2RY13, and CYP27A1 were significantly differently expressed in both PC9 (A) and H1975 (B) cells. [file Image_5.tif]

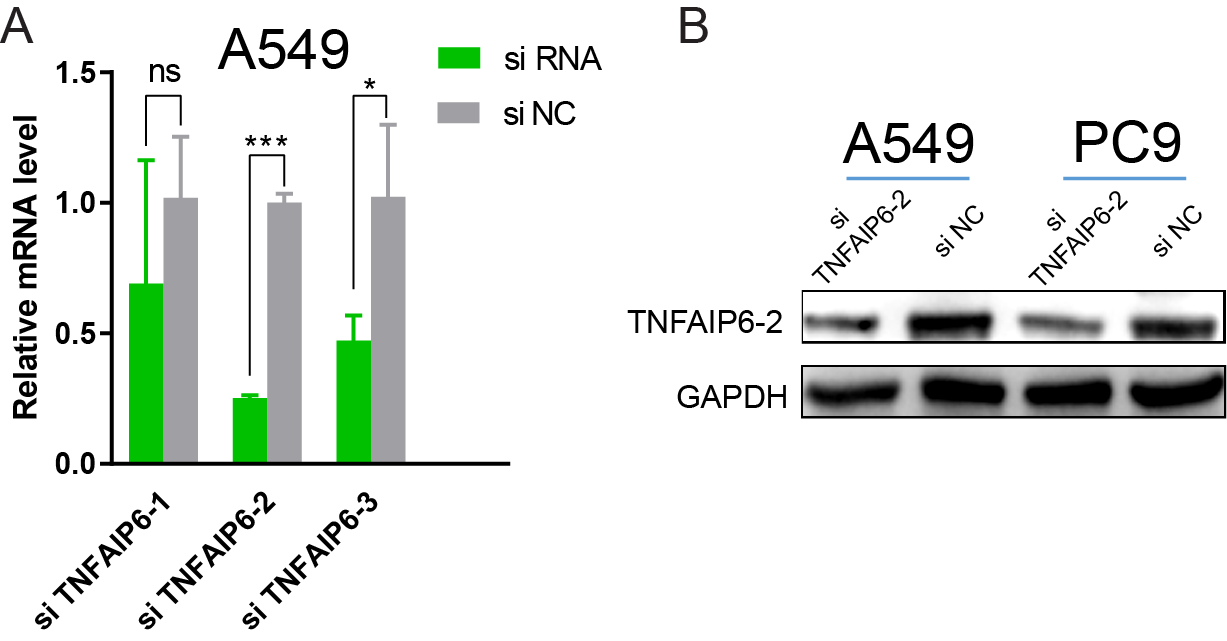

Supplement: Supplementary Figure 6 — Knocking down TNFAIP6 in A549 and PC9. (A) Si TNFAIP6-2 has strongly inhibited the mRNA expression in A549 cells; (B) Si TNFAIP6-2 was selected to transfect into A549 and PC9 and validated by WB. [file Image_6.tif]

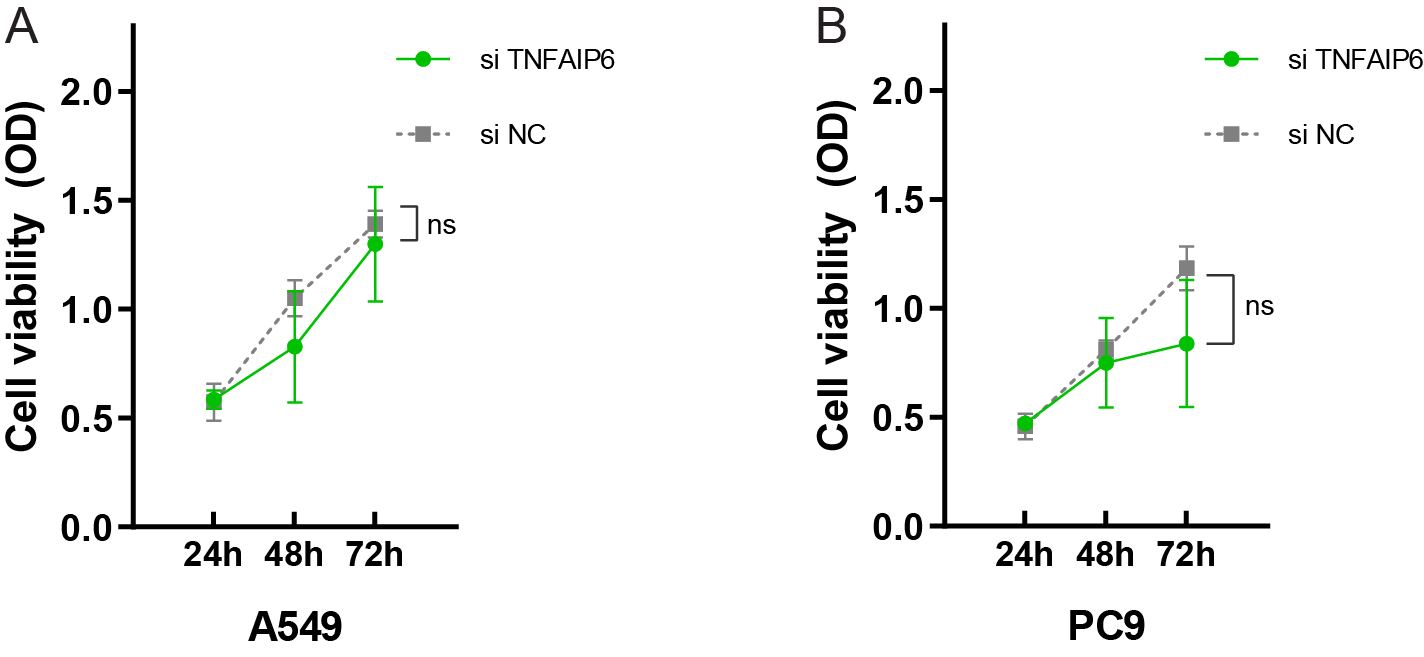

Supplement: Supplementary Figure 7 — CCK8 assay. TNFAIP6 might not affect the proliferation of A549 (A) and PC9 (B) cells. [file Image_7.tif]

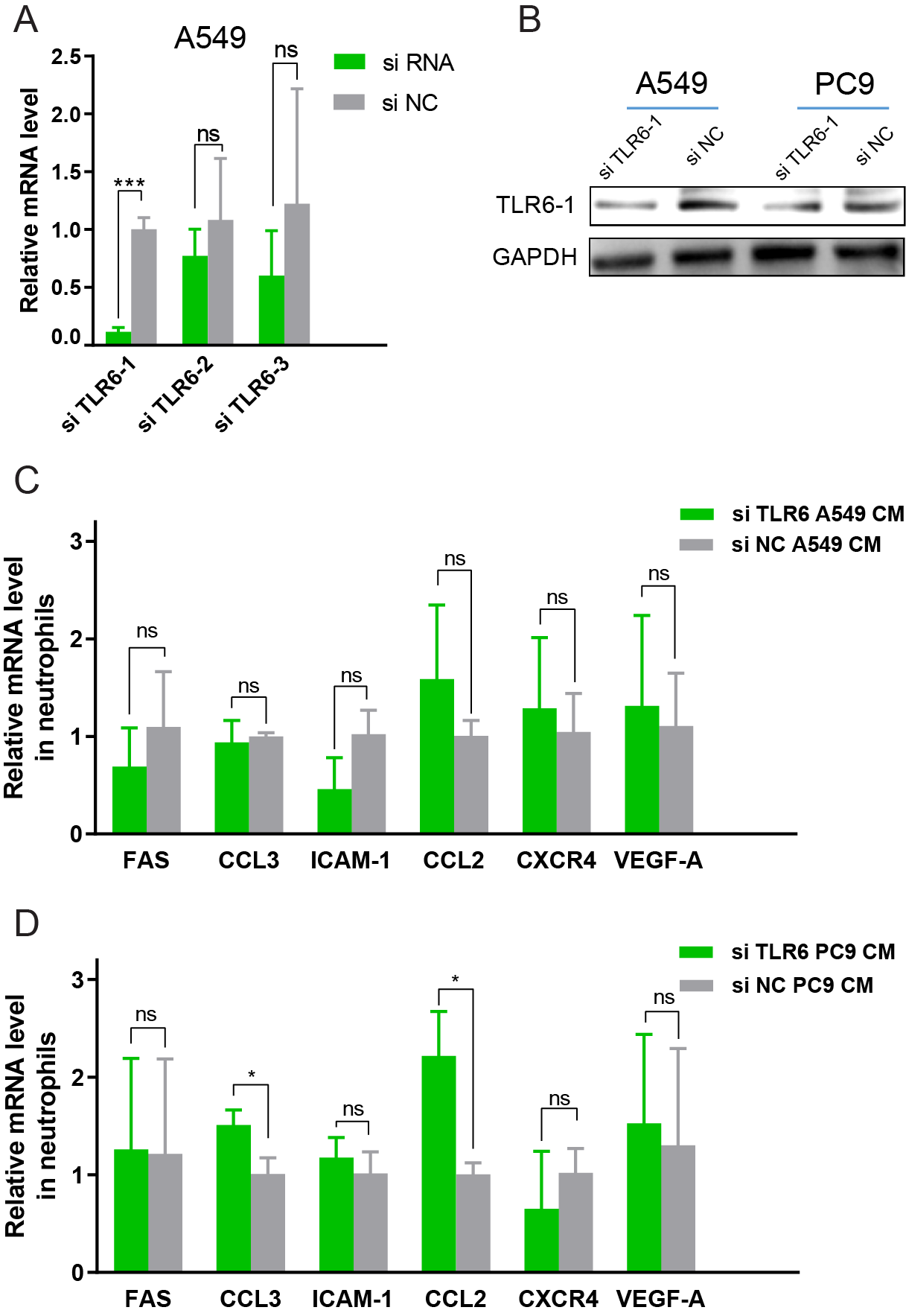

Supplement: Supplementary Figure 8 — TLR6 in lung adenocarcinoma cells might not affect the polarization of neutrophils. (A) Si TLR6-1 strongly inhibited the mRNA expression in A549 cells; (B) Si TLR6-1 was selected to transfect into A549 and PC9 and validated by WB; (C) Knocking down TLR6 in A549 did not affect the expression of FAS, CCL3, ICAM-1, CCL2, CXCR4, and VEGF-A in neutrophils; (D) Although knocking down TLR6 in PC9 unregulated the expression of CCL3 (indicating “N1” polarization), it also elevated the expression of CCL2 (indicating “N2” polarization) in neutrophils. Meanwhile, no significant difference was observed in the expression of FAS, ICAM-1, CXCR4, and VEGF-A. [file Image_8.tif]
