# Supplementary material for: Neutrophil infiltration associated genes on the prognosis and tumor immune microenvironment of lung adenocarcinoma
Source: Front Immunol. 2023 Dec 22;14:1304529. doi: 10.3389/fimmu.2023.1304529 (PMC10777728; doi:10.3389/fimmu.2023.1304529)
Supplement: Supplementary file 9 [file Table_1.docx]

| Case No. | Age(y) | Gender | Smoking status | Pathological diagnosis | Surgical procedure | T stage | N stage | M stage | TNM staging |
| --- | --- | --- | --- | --- | --- | --- | --- | --- | --- |
| 1 | 67 | Male | Never | Adenocarcinoma | Wedge | T1a | N0 | M0 | IA1 |
| 2 | 62 | Female | Never | Adenocarcinoma | Wedge | T1a | N0 | M0 | IA1 |
| 3 | 67 | Female | Never | Adenocarcinoma | Wedge | T1a | N0 | M0 | IA1 |
| 4 | 68 | Male | Current | Adenocarcinoma | Lobectomy | T2a | N0 | M0 | IB |
| 5 | 54 | Female | Never | Adenocarcinoma | Lobectomy | T2a | N0 | M0 | IB |
| 6 | 64 | Male | Current | Adenocarcinoma | Lobectomy | T1a | N0 | M0 | IA1 |
| 7 | 65 | Male | Current | Adenocarcinoma | Lobectomy | T1c | N2 | M0 | IIIA |
| 8 | 68 | Female | Never | Adenocarcinoma | Lobectomy | T1b | N0 | M0 | IA2 |
| 9 | 63 | Female | Never | Adenocarcinoma | Wedge | T1b | N0 | M0 | IA2 |
| 10 | 61 | Female | Current | Adenocarcinoma | Wedge | T1c | N0 | M0 | IA3 |

Supplementary Table 1 Basic clinical information of the ten patients
