# Supplementary material for: Neutrophil infiltration associated genes on the prognosis and tumor immune microenvironment of lung adenocarcinoma
Source: Front Immunol. 2023 Dec 22;14:1304529. doi: 10.3389/fimmu.2023.1304529 (PMC10777728; doi:10.3389/fimmu.2023.1304529)
Supplement: Supplementary file 10 [file Table_2.docx]

Supplementary Table 2 Primers in this study

| Target genes | Primers(5'-3') |
| --- | --- |
| ACTIN-F | TCGTGCGTGACATTAAGGAGAAGC |
| ACTIN-R | CAGGAAGGAAGGCTGGAAGAGTG |
| ALPL-F | GCTGTAAGGACATCGCCTACCA |
| ALPL-R | CCTGGCTTTCTCGTCACTCTCA |
| C4BPA-F | AGTTGGCTGGAGTCATCCTCTC |
| C4BPA-R | GCTGTAGGTGACAGAAAAGCCG |
| CCL2-F | AGAATCACCAGCAGCAAGTGTCC |
| CCL2-R | TCCTGAACCCACTTCTGCTTGG |
| CCL3-F | ACTTTGAGACGAGCAGCCAGTG |
| CCL3-R | TTTCTGGACCCACTCCTCACTG |
| CFP-F | GAATGGGCAGTGCTCTGGAAAG |
| CFP-R | TTGGAGCAGGTGACAGAGCAAG |
| CXCR4-F | CTCCTCTTTGTCATCACGCTTCC |
| CXCR4-R | GGATGAGGACACTGCTGTAGAG |
| CYP27A1-F | GTGCTGCCTTTCTGGAAGCGAT |
| CYP27A1-R | TAGCCAGACACCTGGATGCCAT |
| DDX58-F | CACCTCAGTTGCTGATGAAGGC |
| DDX58-R | GTCAGAAGGAAGCACTTGCTACC |
| DPEP2-F | GTGTGCAACAGTGCTCGGAATG |
| DPEP2-R | CAGTGGACACATTGGCTGATGG |
| FAS-F | GGACCCAGAATACCAAGTGCAG |
| FAS-R | GTTGCTGGTGAGTGTGCATTCC |
| FOSL2-F | AAGAGGAGGAGAAGCGTCGCAT |
| FOSL2-R | GCTCAGCAATCTCCTTCTGCAG |
| GPX3-F | TACGGAGCCCTCACCATTGATG |
| GPX3-R | CAGACCGAATGGTGCAAGCTCT |
| ICAM1-F | AGCGGCTGACGTGTGCAGTAAT |
| ICAM1-R | TCTGAGACCTCTGGCTTCGTCA |
| ITGA5-F | GCCGATTCACATCGCTCTCAAC |
| ITGA5-R | GTCTTCTCCACAGTCCAGCAAG |
| ITPRIP-F | AGAACCTGCTGTGTGCCACAGA |
| ITPRIP-R | CAGGTCGAACTCGTACTTGTGG |
| KIAA0825-F | GCGTCAGAGTTTGTGCTACAAGC |
| KIAA0825-R | TAGGAGGCAGAATGGTCCAGAG |
| MBOAT2-F | GGGTATGACGAAAATGGAGCAGC |
| MBOAT2-R | CTTTTGAGCCAAAGAGCTGTCTG |
| MCTP1-F | GGACAAAGATGCTGGGAAAAGGG |
| MCTP1-R | CTGATGCTGTCAGAGTGACCAG |
| MXD1-F | ACCTGAAGAGGCAGCTGGAGAA |
| MXD1-R | AGATAGTCCGTGCTCTCCACGT |
| NAMPT-F | AGGGTTACAAGTTGCTGCCACC |
| NAMPT-R | CTCCACCAGAACCGAAGGCAAT |
| NCF1-F | AATGGCAGGACCTGTCGGAGAA |
| NCF1-R | CCTGTTCTCTGGATTGATCGCC |
| P2RY13-F | GCCGACTTGATAATGACACTCATG |
| P2RY13-R | CCTAACAGCACGATGCCCACAT |
| PCSK5-F | TGTGGAGAGCACAGACCGACAA |
| PCSK5-R | ACAACGACGTGCTCCAGGTAGT |
| PLAUR-F | CCACTCAGAGAAGACCAACAGG |
| PLAUR-R | GTAACGGCTTCGGGAATAGGTG |
| PRAM1-F | CCGTGGATATGCAGAGCTTTCG |
| PRAM1-R | GGGTTCCACATCGTCATACAGC |
| RELL1-F | GCCCACTAACAAGTCCAGAGAG |
| RELL1-R | CAGACATCAGGCTTCTCCGTTC |
| RGS2-F | CTCTACTCCTGGGAAGCCCAAA |
| RGS2-R | TTGCTGGCTAGCAGCTCGTCAA |
| RNF175-F | GTGCTGGTTCAGTGGAGACAGA |
| RNF175-R | CCCACATAGACAGAAACCGCCA |
| SCARF1-F | AGCTACCGTGTCCAGGATGAAG |
| SCARF1-R | GGCTCGATGAAGCTGTGGTTGA |
| SLC2A14-F | CAATCGGCTCTTTCCAGTTTGGC |
| SLC2A14-R | CAAGGACCAGAGATTCGTGAGC |
| SOD2-F | CTGGACAAACCTCAGCCCTAAC |
| SOD2-R | AACCTGAGCCTTGGACACCAAC |
| TLR2-F | CTTCACTCAGGAGCAGCAAGCA |
| TLR2-R | ACACCAGTGCTGTCCTGTGACA |
| TLR6-F | ACTGACCTTCCTGGATGTGGCA |
| TLR6-R | TGACCTCATCTTCTGGCAGCTC |
| TMEM130-F | GCTCCTATCTCACTAAGACCGTC |
| TMEM130-R | CACGGAGTCTTCAGTCACCATC |
| TNFAIP6-F | TCACCTACGCAGAAGCTAAGGC |
| TNFAIP6-R | TCCAACTCTGCCCTTAGCCATC |
| VEGFA-F | GCCACTACTGTGCCTTTGAGTC |
| VEGFA-R | CCCTCAGAGAATCGCCAGTACT |
