# Supplementary material for: Neutrophil infiltration associated genes on the prognosis and tumor immune microenvironment of lung adenocarcinoma
Source: Front Immunol. 2023 Dec 22;14:1304529. doi: 10.3389/fimmu.2023.1304529 (PMC10777728; doi:10.3389/fimmu.2023.1304529)
Supplement: Supplementary file 11 [file Table_3.docx]

Supplementary Table 3 Univariate Cox regression analysis of the other 257 differentially expressed genes (DEGs) associated with PFS

| DEGs | Hazard ratio (HR) | HR 0.95L | HR 0.95H | P-value |
| --- | --- | --- | --- | --- |
| HRH1 | 1.239183 | 1.023887 | 1.499751 | 0.027638 |
| VGLL3 | 1.15819 | 1.028078 | 1.304769 | 0.015718 |
| IL1A | 1.163504 | 1.044564 | 1.295988 | 0.005916 |
| ZC3H12C | 1.297354 | 1.075612 | 1.564809 | 0.006486 |
| CCR2 | 0.866416 | 0.758255 | 0.990005 | 0.035065 |
| MS4A2 | 0.841369 | 0.736121 | 0.961665 | 0.011301 |
| SLC18A2 | 0.815797 | 0.685052 | 0.971494 | 0.022344 |
| SIGLEC6 | 0.81018 | 0.66268 | 0.990511 | 0.040077 |
| PROS1 | 1.174816 | 1.027111 | 1.343762 | 0.018765 |
| CPA3 | 0.914242 | 0.846714 | 0.987156 | 0.022013 |
| HLA-DRA | 0.879928 | 0.798085 | 0.970165 | 0.010227 |
| ANO6 | 1.234095 | 1.021495 | 1.490942 | 0.029226 |
| KLF10 | 1.238949 | 1.019096 | 1.506232 | 0.031575 |
| TNFSF13 | 0.836453 | 0.715176 | 0.978296 | 0.025449 |
| LINC02605 | 1.251967 | 1.010815 | 1.550651 | 0.039542 |
| HLA-DPA1 | 0.892663 | 0.812953 | 0.980189 | 0.017348 |
| HLA-DMB | 0.870157 | 0.778608 | 0.972469 | 0.0142 |
| NABP1 | 1.166486 | 1.00583 | 1.352802 | 0.041665 |
| GNB4 | 1.207523 | 1.032343 | 1.41243 | 0.018374 |
| LINC01936 | 0.825342 | 0.708105 | 0.96199 | 0.014061 |
| HPGDS | 0.848674 | 0.756114 | 0.952564 | 0.005357 |
| SP110 | 1.343086 | 1.004776 | 1.795306 | 0.046356 |
| BIRC3 | 1.181892 | 1.057906 | 1.32041 | 0.003122 |
| CX3CR1 | 0.858099 | 0.758919 | 0.97024 | 0.014603 |
| PHLDB2 | 1.205472 | 1.05659 | 1.375332 | 0.005463 |
| TRAV8-1 | 0.789769 | 0.631305 | 0.988009 | 0.038872 |
| KCNA3 | 0.886075 | 0.78775 | 0.996672 | 0.043851 |
| XCR1 | 0.777617 | 0.62408 | 0.968928 | 0.025011 |
| ADCY9 | 0.849708 | 0.73399 | 0.983669 | 0.029227 |
| SIGLEC17P | 0.730999 | 0.599221 | 0.891757 | 0.002005 |
| ACSL4 | 1.211232 | 1.045911 | 1.402685 | 0.010483 |
| RASAL2 | 1.246635 | 1.039075 | 1.495655 | 0.017669 |
| ITGAV | 1.157942 | 1.006266 | 1.332479 | 0.040642 |
| NAMPTP1 | 1.126049 | 1.021067 | 1.241825 | 0.017431 |
| LAMA4-AS1 | 1.765843 | 1.076274 | 2.89722 | 0.02439 |
| GCSAML | 0.741527 | 0.563885 | 0.975132 | 0.032339 |
| HLA-DPB1 | 0.879836 | 0.796447 | 0.971955 | 0.01174 |
| HAS2 | 1.137954 | 1.006402 | 1.286702 | 0.03923 |
| TESPA1 | 0.830162 | 0.69258 | 0.995076 | 0.044077 |
| TICAM2 | 2.386399 | 1.049007 | 5.428849 | 0.038075 |
| LOX | 1.193991 | 1.065736 | 1.33768 | 0.002228 |
| SAMD9 | 1.14737 | 1.01158 | 1.301388 | 0.032426 |
| SERPIND1 | 0.903553 | 0.840489 | 0.971349 | 0.006006 |
| HLA-DRB1 | 0.878861 | 0.800554 | 0.964829 | 0.006689 |
| MAP3K20 | 1.248562 | 1.039882 | 1.499121 | 0.017356 |
| FUCA1 | 0.80768 | 0.68411 | 0.95357 | 0.011697 |
| NCEH1 | 1.167986 | 1.003095 | 1.359982 | 0.045527 |
| SCN4B | 0.827451 | 0.714813 | 0.957837 | 0.011182 |
| NEDD4 | 1.283954 | 1.074056 | 1.534873 | 0.006062 |
| LINC01537 | 1.955533 | 1.35381 | 2.824701 | 0.000351 |
| CD101 | 0.821914 | 0.683854 | 0.987847 | 0.036592 |
| CCL13 | 0.907946 | 0.83927 | 0.982241 | 0.016107 |
| SLC15A2 | 0.86792 | 0.775112 | 0.971839 | 0.014088 |
| TRHDE | 1.400018 | 1.065584 | 1.839415 | 0.015689 |
| CD74 | 0.877325 | 0.796477 | 0.966379 | 0.007971 |
| STING1 | 0.862018 | 0.75164 | 0.988605 | 0.033678 |
| CELF2-AS1 | 1.276074 | 1.036273 | 1.571368 | 0.021707 |
| DSEL | 1.178874 | 1.006155 | 1.381243 | 0.041762 |
| HLA-DMA | 0.830992 | 0.741012 | 0.931899 | 0.001545 |
| TPSAB1 | 0.893697 | 0.819317 | 0.974829 | 0.011246 |
| AL157895.2 | 0.728285 | 0.551586 | 0.961587 | 0.025337 |
| CRIM1 | 1.229043 | 1.064138 | 1.419502 | 0.005021 |
| FLT1 | 1.195051 | 1.028802 | 1.388165 | 0.019727 |
| CYP4F22 | 0.651705 | 0.430376 | 0.986855 | 0.043128 |
| LATS2 | 1.323379 | 1.076773 | 1.626464 | 0.007745 |
| MFAP4 | 0.909366 | 0.833586 | 0.992034 | 0.032345 |
| NAPSB | 0.878397 | 0.785993 | 0.981664 | 0.022237 |
| CLEC10A | 0.853151 | 0.760401 | 0.957215 | 0.006838 |
| INHBA | 1.133345 | 1.02273 | 1.255923 | 0.016898 |
| GLRX | 1.232685 | 1.056593 | 1.438123 | 0.007816 |
| TMEM64 | 1.226366 | 1.05673 | 1.423234 | 0.007223 |
| JPH4 | 0.655559 | 0.462787 | 0.928629 | 0.017467 |
| DMBT1 | 0.94535 | 0.896014 | 0.997402 | 0.03987 |
| RAB27B | 1.157017 | 1.042592 | 1.284 | 0.006051 |
| HLA-DQB2 | 0.905715 | 0.841437 | 0.974904 | 0.008372 |
| FCER1A | 0.913402 | 0.844358 | 0.988091 | 0.023901 |
| RHOXF1-AS1 | 0.874235 | 0.774526 | 0.98678 | 0.029603 |
| SELENOP | 0.890548 | 0.800411 | 0.990836 | 0.03325 |
| RELN | 1.160448 | 1.002942 | 1.342688 | 0.045561 |
| NOTCH2NLA | 1.387665 | 1.010236 | 1.906103 | 0.043089 |
| RTN1 | 0.832105 | 0.711944 | 0.972547 | 0.0209 |
| HIF1A | 1.252378 | 1.063213 | 1.4752 | 0.007068 |
| ATP13A4 | 0.904547 | 0.836703 | 0.977893 | 0.011671 |
| NT5C1B | 1.47845 | 1.037982 | 2.105832 | 0.030271 |
| CD109 | 1.168901 | 1.058759 | 1.290501 | 0.001997 |
| LINC00189 | 1.259528 | 1.011066 | 1.569049 | 0.039578 |
| KLHL4 | 1.270168 | 1.043381 | 1.546248 | 0.017165 |
| HAS3 | 0.909653 | 0.836555 | 0.989138 | 0.026727 |
| GFPT2 | 1.200993 | 1.05364 | 1.368952 | 0.0061 |
| LINC00996 | 0.811381 | 0.662313 | 0.994001 | 0.043585 |
| PCDH7 | 1.151441 | 1.04622 | 1.267245 | 0.003926 |
| AC104966.1 | 1.149568 | 1.016647 | 1.299867 | 0.026195 |
| RHEX | 0.845941 | 0.726368 | 0.985198 | 0.031417 |
| CTSG | 0.845308 | 0.744215 | 0.960135 | 0.009711 |
| GPRIN3 | 1.199185 | 1.029723 | 1.396536 | 0.019451 |
| TMEM52 | 0.863919 | 0.751328 | 0.993381 | 0.040056 |
| SCN2B | 0.726042 | 0.553327 | 0.952668 | 0.020899 |
| CSGALNACT1 | 1.289926 | 1.102829 | 1.508764 | 0.001452 |
| PRELP | 0.893784 | 0.811286 | 0.984671 | 0.023051 |
| HLA-V | 1.207946 | 1.044448 | 1.397038 | 0.010895 |
| AC021106.1 | 1.40784 | 1.144453 | 1.731843 | 0.001209 |
| GSDME | 1.178984 | 1.024079 | 1.35732 | 0.021961 |
| LINC01150 | 0.765862 | 0.632974 | 0.926648 | 0.00608 |
| CENPM | 1.145673 | 1.000966 | 1.3113 | 0.048384 |
| CLMP | 1.132484 | 1.006918 | 1.273709 | 0.037992 |
| RAB44 | 0.567271 | 0.367796 | 0.874932 | 0.010338 |
| CD40LG | 0.82945 | 0.712038 | 0.966224 | 0.016342 |
| AP001885.1 | 1.298797 | 1.015596 | 1.660967 | 0.037225 |
| FAT3 | 1.327176 | 1.008445 | 1.746645 | 0.043386 |
| AQP4 | 0.930797 | 0.875846 | 0.989196 | 0.020897 |
| THSD7A | 1.220573 | 1.052813 | 1.415066 | 0.008237 |
| FRRS1 | 1.178603 | 1.006449 | 1.380204 | 0.041375 |
| AL049840.7 | 1.178223 | 1.011805 | 1.372012 | 0.03477 |
| TPSB2 | 0.8643 | 0.79909 | 0.934831 | 0.000269 |
| ITGB1 | 1.253402 | 1.068389 | 1.470454 | 0.005575 |
| GSN-AS1 | 1.285805 | 1.028861 | 1.606917 | 0.027098 |
| VCAN | 1.118535 | 1.016381 | 1.230956 | 0.021878 |
| AC110995.1 | 0.747479 | 0.56145 | 0.995148 | 0.046231 |
| SLC26A2 | 1.163051 | 1.029645 | 1.313743 | 0.015101 |
| FSTL1 | 1.151527 | 1.011021 | 1.311559 | 0.033582 |
| LINC02273 | 0.690135 | 0.507604 | 0.938303 | 0.017968 |
| ADAMTS8 | 0.858036 | 0.757969 | 0.971314 | 0.01552 |
| AC022150.4 | 1.210966 | 1.028873 | 1.425287 | 0.021317 |
| AP000919.2 | 1.252982 | 1.039713 | 1.509998 | 0.017833 |
| KRT18P31 | 1.409618 | 1.040081 | 1.910451 | 0.026876 |
| RRAD | 0.907444 | 0.836309 | 0.98463 | 0.019708 |
| CASP12 | 0.661386 | 0.43938 | 0.995564 | 0.047561 |
| CD1C | 0.88359 | 0.802214 | 0.97322 | 0.012052 |
| P2RX1 | 0.812246 | 0.679993 | 0.970222 | 0.021827 |
| ADGRF4 | 1.151663 | 1.057282 | 1.254468 | 0.001209 |
| AC127502.1 | 1.983594 | 1.326014 | 2.967271 | 0.000858 |
| NTRK1 | 0.65167 | 0.43684 | 0.972151 | 0.035873 |
| SEMA3C | 1.155503 | 1.031864 | 1.293957 | 0.012308 |
| UBE2S | 1.15317 | 1.013688 | 1.311844 | 0.030261 |
| MN1 | 1.17688 | 1.019824 | 1.358123 | 0.025843 |
| RSPO3 | 1.117361 | 1.031721 | 1.21011 | 0.006381 |
| SUCLG2-AS1 | 1.353129 | 1.023025 | 1.78975 | 0.034048 |
| GFRA2 | 0.735634 | 0.544465 | 0.993924 | 0.045537 |
| MRO | 1.530447 | 1.113494 | 2.10353 | 0.008731 |
| HAS2-AS1 | 1.257296 | 1.000257 | 1.580389 | 0.049744 |
| FAM126A | 1.157 | 1.009819 | 1.325633 | 0.035666 |
| MEDAG | 1.124995 | 1.015567 | 1.246215 | 0.024082 |
| DCBLD2 | 1.118903 | 1.002102 | 1.249318 | 0.045793 |
| GATA1 | 0.533833 | 0.342281 | 0.832582 | 0.005641 |
| SORCS2 | 0.895334 | 0.816355 | 0.981953 | 0.018951 |
| MYO1E | 1.317847 | 1.126778 | 1.541315 | 0.000553 |
| IRF4 | 0.88506 | 0.786853 | 0.995523 | 0.041878 |
| PLXNA4 | 1.300458 | 1.080738 | 1.564848 | 0.005398 |
| HLA-DRB5 | 0.88082 | 0.815566 | 0.951296 | 0.001232 |
| AC037198.1 | 1.128163 | 1.008426 | 1.262116 | 0.035158 |
| EFNB2 | 1.174191 | 1.036949 | 1.329598 | 0.011339 |
| AL022334.2 | 1.877207 | 1.188009 | 2.966229 | 0.006976 |
| INMT | 0.901597 | 0.823536 | 0.987057 | 0.024967 |
| MYH15 | 1.305192 | 1.028119 | 1.656935 | 0.028689 |
| KRT8P37 | 1.521205 | 1.018036 | 2.27307 | 0.040639 |
| TREML1 | 0.749806 | 0.596044 | 0.943234 | 0.01393 |
| NR2F2-AS1 | 1.434064 | 1.071399 | 1.919491 | 0.015368 |
| LINC00892 | 0.748153 | 0.578356 | 0.967801 | 0.027164 |
| MFSD4A | 0.915249 | 0.846316 | 0.989797 | 0.026646 |
| HLA-DQB1-AS1 | 0.856834 | 0.77691 | 0.944981 | 0.001984 |
| TTC9 | 1.240326 | 1.096522 | 1.40299 | 0.000614 |
| LINC02201 | 1.680332 | 1.017136 | 2.775946 | 0.042734 |
| SERPINH1P1 | 2.058775 | 1.038439 | 4.081659 | 0.038642 |
| CX3CL1 | 0.905371 | 0.831748 | 0.985511 | 0.021605 |
| HSD17B6 | 0.899197 | 0.82579 | 0.979129 | 0.01447 |
| AC133963.1 | 0.831041 | 0.731655 | 0.943926 | 0.0044 |
| SFTA1P | 0.925075 | 0.865949 | 0.988237 | 0.020827 |
| SNAI2 | 1.183226 | 1.054889 | 1.327176 | 0.004076 |
| SF3A3P2 | 1.476618 | 1.012384 | 2.153728 | 0.042983 |
| TVP23A | 1.22942 | 1.002589 | 1.507572 | 0.047166 |
| PADI6 | 0.012396 | 0.000705 | 0.218062 | 0.002691 |
| AGER | 0.91884 | 0.859757 | 0.981984 | 0.012557 |
| RAI2 | 0.858643 | 0.746789 | 0.987252 | 0.032344 |
| LINC02256 | 1.441232 | 1.085944 | 1.91276 | 0.011378 |
| SPC24 | 1.207047 | 1.058694 | 1.37619 | 0.004917 |
| H2AC20 | 1.15373 | 1.033918 | 1.287425 | 0.010582 |
| HLA-DQB1 | 0.890012 | 0.818769 | 0.967454 | 0.006196 |
| PXDN | 1.132229 | 1.017573 | 1.259804 | 0.022622 |
| LAMC2 | 1.129322 | 1.034517 | 1.232814 | 0.006558 |
| PMEPA1 | 1.206582 | 1.064502 | 1.367625 | 0.003305 |
| DDX18P1 | 1.332224 | 1.053855 | 1.684122 | 0.016459 |
| TMPRSS2 | 0.900007 | 0.823547 | 0.983567 | 0.020032 |
| AP001528.1 | 1.295852 | 1.047873 | 1.602514 | 0.016781 |
| C1orf116 | 0.921483 | 0.854677 | 0.993511 | 0.033213 |
| SNX30 | 0.858573 | 0.756535 | 0.974375 | 0.018172 |
| CD5 | 0.86625 | 0.76371 | 0.982556 | 0.025501 |
| SYT16 | 1.266456 | 1.027413 | 1.561116 | 0.026873 |
| KLHDC7A | 0.869044 | 0.782479 | 0.965185 | 0.008745 |
| FHL2 | 1.13795 | 1.022915 | 1.265921 | 0.017471 |
| FOXG1 | 1.216747 | 1.013815 | 1.4603 | 0.035088 |
| COL5A1 | 1.106364 | 1.001743 | 1.221912 | 0.046116 |
| TDGF1P7 | 1.637908 | 1.008517 | 2.660085 | 0.046126 |
| AC108058.1 | 1.192929 | 1.009972 | 1.40903 | 0.037823 |
| ADAMTS7P3 | 0.774507 | 0.621925 | 0.964522 | 0.022451 |
| LDLRAD2 | 0.77217 | 0.606823 | 0.98257 | 0.035467 |
| PIGR | 0.946349 | 0.90176 | 0.993143 | 0.025132 |
| LINC01322 | 1.44307 | 1.18768 | 1.753377 | 0.000224 |
| SERPINE1 | 1.158065 | 1.05257 | 1.274132 | 0.002601 |
| POSTN | 1.125776 | 1.022884 | 1.239018 | 0.015408 |
| NID2 | 1.219378 | 1.072086 | 1.386907 | 0.00253 |
| PAPPA | 1.160353 | 1.001314 | 1.344652 | 0.047993 |
| CCL20 | 1.112979 | 1.042997 | 1.187658 | 0.001236 |
| SFTPC | 0.963309 | 0.930292 | 0.997498 | 0.035664 |
| TRAV10 | 0.790676 | 0.625308 | 0.999776 | 0.049782 |
| NPAS2 | 1.218249 | 1.05941 | 1.400902 | 0.005612 |
| VEGFD | 0.900436 | 0.820359 | 0.988329 | 0.027313 |
| PPIAP39 | 0.858199 | 0.74816 | 0.984423 | 0.028947 |
| NRGN | 0.907623 | 0.832507 | 0.989515 | 0.027871 |
| TRAV8-4 | 0.826054 | 0.691456 | 0.986852 | 0.035221 |
| AC245041.2 | 1.148661 | 1.016698 | 1.297751 | 0.026019 |
| RCAN2 | 0.846333 | 0.753416 | 0.950709 | 0.004926 |
| C11orf21 | 0.782883 | 0.637705 | 0.961111 | 0.019336 |
| PLN | 1.110314 | 1.000201 | 1.23255 | 0.04956 |
| VAX1 | 1.856767 | 1.378156 | 2.501593 | 4.72E-05 |
| NID1 | 1.159513 | 1.020127 | 1.317944 | 0.023517 |
| AC112694.1 | 1.422071 | 1.006947 | 2.008333 | 0.045579 |
| AC025580.2 | 1.386964 | 1.126333 | 1.707905 | 0.002069 |
| OLFM1 | 0.888059 | 0.790593 | 0.997541 | 0.045342 |
| ADARB2-AS1 | 1.377086 | 1.007654 | 1.881961 | 0.044664 |
| COL5A2 | 1.124264 | 1.018479 | 1.241036 | 0.020172 |
| SH3TC2 | 1.466691 | 1.025371 | 2.097957 | 0.035981 |
| DLG2 | 1.328676 | 1.023229 | 1.725304 | 0.032985 |
| AC079313.1 | 1.403517 | 1.11134 | 1.772508 | 0.004422 |
| KLF4 | 1.115795 | 1.002667 | 1.241687 | 0.044559 |
| NFIX | 0.876864 | 0.784045 | 0.980673 | 0.021344 |
| CDCA3 | 1.156129 | 1.015314 | 1.316473 | 0.028574 |
| HSPD1P7 | 1.717971 | 1.067791 | 2.764047 | 0.025728 |
| PLD4 | 0.835951 | 0.711746 | 0.981831 | 0.029007 |
| AC011005.4 | 1.350179 | 1.074596 | 1.696435 | 0.009948 |
| BTNL9 | 0.869729 | 0.75771 | 0.99831 | 0.047254 |
| TROAP | 1.118684 | 1.008553 | 1.240842 | 0.03392 |
| AC093724.1 | 1.244417 | 1.022424 | 1.514611 | 0.029171 |
| TARID | 1.430088 | 1.148514 | 1.780693 | 0.001385 |
| LDHAP5 | 1.368929 | 1.122045 | 1.670136 | 0.001969 |
| SFTPD | 0.932904 | 0.883525 | 0.985043 | 0.012312 |
| TLR8-AS1 | 0.826948 | 0.713625 | 0.958267 | 0.011509 |
| AC020763.3 | 1.550713 | 1.11219 | 2.162139 | 0.009682 |
| FRMD6 | 1.16744 | 1.027007 | 1.327076 | 0.01791 |
| SLC47A1 | 0.837357 | 0.744377 | 0.941951 | 0.003119 |
| NAPSA | 0.9201 | 0.875647 | 0.96681 | 0.000981 |
| IRX6 | 0.883411 | 0.79698 | 0.979214 | 0.018284 |
| DNASE1L3 | 0.852331 | 0.738777 | 0.98334 | 0.028506 |
| GLI2 | 1.316053 | 1.094338 | 1.582687 | 0.003526 |
| SNORA80E | 1.151553 | 1.018232 | 1.302331 | 0.024591 |
| CDKL2 | 0.881079 | 0.792411 | 0.979669 | 0.019309 |
| SERPINA3 | 1.201686 | 1.023934 | 1.410296 | 0.024477 |
| FAM189A2 | 0.863379 | 0.770646 | 0.967269 | 0.011277 |
| TRAV17 | 0.831924 | 0.696173 | 0.994147 | 0.042913 |
| GGTLC1 | 0.909373 | 0.854246 | 0.968059 | 0.002907 |
| TPSD1 | 0.878662 | 0.793774 | 0.972627 | 0.012582 |
| ADAMTS15 | 1.222823 | 1.052797 | 1.420308 | 0.00845 |
| FOSL1 | 1.090401 | 1.004917 | 1.183156 | 0.037735 |
| ZNF737 | 0.846967 | 0.727824 | 0.985614 | 0.031769 |
| CRTAC1 | 0.915863 | 0.848624 | 0.988431 | 0.023878 |
| CP | 1.076133 | 1.002022 | 1.155726 | 0.043858 |
| GALNT4 | 1.235383 | 1.001884 | 1.523302 | 0.047977 |
| AC012065.1 | 1.664077 | 1.257002 | 2.202982 | 0.000374 |
